# Modulation of the splicing regulatory function of SRSF10 by a novel compound that impairs HIV-1 replication

**DOI:** 10.1093/nar/gkw1223

**Published:** 2016-12-07

**Authors:** Lulzim Shkreta, Marco Blanchette, Johanne Toutant, Emmanuelle Wilhelm, Brendan Bell, Benjamin A. Story, Ahalya Balachandran, Alan Cochrane, Peter K. Cheung, P. Richard Harrigan, David S. Grierson, Benoit Chabot

**Affiliations:** 1 Department of Microbiology and Infectious Diseases, Faculty of Medicine and Health Sciences, Université de Sherbrooke, Sherbrooke, QC, J1E 4K8, Canada; 2 Stowers Institute for Medical Research, Kansas City, MO 64110, USA; 3 Department of Molecular Genetics, University of Toronto, Toronto, ON, M5S 1A8, Canada; 4 BC Centre for Excellence in HIV/AIDS, 608–1081 Burrard Street, Vancouver, BC, V6Z 1Y6, Canada; 5 Department of Medicine, University of British Columbia, Vancouver, BC, V6T 1Z3, Canada; 6 Faculty of Pharmaceutical Sciences, University of British Columbia, Vancouver, BC, V6T 1Z3, Canada

## Abstract

We recently identified the 4-pyridinone-benzisothiazole carboxamide compound 1C8 as displaying strong anti-HIV-1 potency against a variety of clinical strains *in vitro*. Here we show that 1C8 decreases the expression of HIV-1 and alters splicing events involved in the production of HIV-1 mRNAs. Although 1C8 was designed to be a structural mimic of the fused tetracyclic indole compound IDC16 that targets SRSF1, it did not affect the splice site shifting activity of SRSF1. Instead, 1C8 altered splicing regulation mediated by SRSF10. Depleting SRSF10 by RNA interference affected viral splicing and, like 1C8, decreased expression of Tat, Gag and Env. Incubating cells with 1C8 promoted the dephosphorylation of SRSF10 and increased its interaction with hTra2β, a protein previously implicated in the control of HIV-1 RNA splicing. While 1C8 affects the alternative splicing of cellular transcripts controlled by SRSF10 and hTra2β, concentrations greater than those needed to inhibit HIV-1 replication were required to elicit significant alterations. Thus, the ability of 1C8 to alter the SRSF10-dependent splicing of HIV-1 transcripts, with minor effects on cellular splicing, supports the view that SRSF10 may be used as a target for the development of new anti-viral agents.

## INTRODUCTION

The efficacy of combination Anti-Retroviral Therapy (cART) is such that HIV-1/AIDS is treated as a chronic infection in developed countries, and aggressive measures are being taken to expand ART to the developing world, where the majority of HIV-infected people reside (1). Despite this progress, recent statistics show that ∼2.4 million new infections occur per year and 1.6 million people die from AIDS (2). The successful treatment of HIV infection in coming years will require, among other things, addressing the inherent limitations of ART, which include strict adherence to treatment schedules, drug toxicity and the emergence of drug resistance (3). Indeed, there continues to be a need to discover new drugs that act through unexploited mechanisms of action that bypass resistance, have minimal toxicity, and address the problem of activating the latent viral pool (4).

Following transcription from the integrated HIV-1 genome, the 9 kb HIV-1 primary transcript is alternatively spliced through the use of four 5΄ splice sites (D1–D4) and eight 3΄ splice sites (A1–A7) to produce 15 viral proteins. Of these, auxiliary proteins such as Tat, Rev and Nef, respectively stimulate HIV-1 transcription, allow transport to the cytoplasm of unspliced and partially spliced transcripts encoding viral enzymes and structural proteins, and enhance virulence in the host (5,6). The production of Tat, Rev and Nef must therefore be tightly controlled, as splicing imbalances can severely compromise HIV-1 replication (7).

A variety of cellular hnRNP and SR proteins have been implicated in HIV-1 pre-mRNA splicing (8,9). Targeting the expression or activity of these splicing regulatory proteins may represent novel anti-HIV-1 strategy. Although hnRNP and SR proteins also regulate the alternative splicing of cellular pre-mRNAs (10,11), the fact that HIV produces over 40 distinct mRNAs by alternative splicing may render its replication exquisitely sensitive to even slight disturbances in the activity of these host factors. To date, attempts at altering HIV-1 splicing have focused on SR proteins. The localization and activity of these proteins are controlled by a variety of kinases, such as the SRPKs and the CLKs (12). Indeed, a small molecule inhibitor of CLKs was shown to alter HIV-1 RNA processing and to inhibit HIV-1 replication (13). Further, digoxin, a drug used to treat congestive heart failure, modulates the activity of CLKs and increases the ability of SRSF3 (aka SRp20) to alter HIV-1 pre-mRNA splicing (14). Pertinent to the current study, the fused tetracyclic indole compound IDC16, which inhibits the function of SRSF1, displays anti-HIV-1 activity (15,16).

Following the discovery of IDC16, we have carried out a diversity-driven library synthesis-screening program to design a series of novel diheteroarylamide-based molecules displaying anti-HIV-1 activity against a variety of clinical strains. Compound 1C8 was identified as the most active compound, and subsequent viability studies revealed that it displayed very low cellular toxicity (17). Given that 1C8 was inspired by the structure of the SRSF1 inhibitor IDC16 (16), we tested the impact of 1C8 on HIV-1 expression, splicing, and the activity of selected SR proteins. In contrast to the results for IDC16, we identify the splicing regulatory protein SRSF10 as being affected by 1C8. Notably, treatment of cells with 1C8 promotes the dephosphorylation of SRSF10 and increases its interaction with hTra2β, a known regulator of HIV-1 splicing (18,19). Consistent with 1C8 targeting the activity of SRSF10 and hTra2β, we identify cellular alternative splicing events controlled by SRSF10 and hTra2β that are reactive to 1C8, but that require concentrations higher than those needed for HIV-1 inhibition. Overall, with its robust effect on HIV-1 replication and minimal impact on cellular gene expression and splicing, 1C8 may represent a novel and innocuous agent for the treatment of HIV-1 infection.

## MATERIALS AND METHODS

### PBMC assays

To examine the effect of compounds on viral replication, studies were carried out in peripheral blood mononuclear cells (PBMCs). PBMCs were isolated from healthy (HIV-uninfected) volunteer blood donors, as described in Dobson-Belaire *et al.* (20). Informed consent was obtained from participants in accordance with the guidelines for conduct of clinical research at the University of Toronto and St. Michael's Hospital, Toronto, Ontario, Canada. Stored PBMCs were thawed, washed with RPMI 1640 complete medium and cultured in RPMI 1640 complete medium containing 2 μg/ml of PHA-L (Sigma-Aldrich) and 20 U/ml of IL-2 (BD Pharmingen) for 72 h. Subsequently, cells were counted and a portion of the cells was separated to another tube for uninfected control treatments. The remaining PBMCs were resuspended in media containing HIV-1 BaL at a multiplicity of infection (MOI) of ∼0.01 and infected by spinoculation, following which cells were washed twice with room temperature RPMI 1640 complete medium and resuspended to a concentration of 5 × 10^5^ cells/ml in complete RPMI 1640 containing 40 U/ml of IL-2. Compounds were added to infected PBMCs or uninfected control PBMCs. Azidothymidine (AZT, Sigma-Aldrich) was used as control treatment at a final concentration of 3.74 μM. On day 4 post-infection, culture medium was replenished with the compounds and IL-2 in fresh complete RPMI 1640 medium. On days 2, 4, 6 and 8 post-infection, culture supernatant was harvested, virus lysed by adjusting to 1% Triton X-100 and stored at −20°C for p24 antigen ELISA. Culture was harvested to assess percent cell viability by trypan blue exclusion using glasstic slides (Kova). Relative percent cell viability in compound treated samples versus DMSO-control treated samples was calculated as follows: (total viable cells/total cells)_compound_/(total viable cells/total cells)_DMSO_. ELISA for Gag-p24 antigen was performed on cell supernatants using kits purchased from XpressBio extended range kit and performed according to manufacturer's instructions.

### RT-PCR assays and northern blot

Quantitative and endpoint RT-PCR analysis was performed by the RNomics Platform at Université de Sherbrooke. The list of primers is provided in Supplementary Table S4. For HIV-1 transcripts, primers were designed based on the complete genome sequence of human immunodeficiency virus 1: NCBI Reference Sequence: NC_001802.1. Design and validation of quantitative RT-PCR assays were as previously described (21,22). A total of 200 ng of RNA (quantitated using the Thermo Scientific NanoDrop) measured for integrity (using the Agilent LabChip station) was reverse transcribed using random hexamers with Transcriptor Reverse transcriptase in a final volume of 10 μl. Ten nanogram of cDNA were used for the quantification in the presence of the specific primers at 0.2 μM in a 10 μl reaction in triplicates. Reactions were carried out in the ABI 7500 qPCR (Applied Biosystems). A first cycle of 10 min at 95°C was followed by 40 cycles of 15 s at 94°C, 20 s at 55°C and 20 s at 68°C. Fluorescence measurement using SYBR Green was performed and values were normalized to the control sample.

For the cellular genes, endpoint analysis was performed using a set of alternative splicing units derived from the RefSeq database. Total RNA was extracted using TRIzol and quantified using a 2100 Bioanalyzer (Agilent Inc.). A total of 2 μg of RNA was reverse transcribed using a mix of random hexamers and oligo (dT) and the Omniscript reverse transcriptase (Qiagen) in a final volume of 20 μl. Twenty nanogram of cDNA were amplified with 0.2 U/10 μl of HotStarTaq DNA Polymerase (Qiagen) in the buffer provided by the manufacturer, and in the presence of the specific primers (IDT) for each splicing unit (at concentrations ranging from 0.3 to 0.6 μM) and dNTPs. Reactions were carried out in the GeneAmp PCR system 9700 (Applied Biosystems). A first cycle of 15 min at 95°C was followed by 35 cycles of 30 s at 94°C, 30 s at 55°C and 1 min at 72°C. Thermocycling was concluded with an extension step of 10 min at 72°C. Visualization and analysis of amplified products were done using the LabChip HT™ DNA assay on a Caliper LC-90 automated microfluidic station (Caliper).

For northern analysis, HeLa-HIV cells were harvested 24 h after treatment or not with 1C8 and total RNA was extracted using TRIzol. Briefly, 10 μg of total RNA was separated on a denaturing 0.8% MOPS–formaldehyde–agarose gel, transferred to a Hybond-N+ nylon membrane (GE Healthcare, Canada) and ultraviolet cross-linked. The membrane was incubated with a HIV-specific ^32^P-labeled probe to visualize viral RNAs and re-incubated with actin-specific ^32^P-labeled probe. The membrane was exposed on a Phosphor screen that was scanned on a STORM PhosphorImager 860 (GE Healthcare). HIV and actin probes were produced by PCR performed in the presence of [α-^32^P]dCTP. The template for the HIV-specific probe was a PCR amplicon of 490 nt generated from RT-PCR using total RNA extracted from HeLa-HIV cells and HIV-specific primers (MS-6-FWD 5΄-TGG AAG CAT CCA GGA AGT CAG-3΄ and MS-4-REV 5΄-CTC AGC TAC TGC TAT GGC TGT G-3΄). Primers used for production of the actin probe were 5΄-TCG TGA TGG ACT CCG GTG AC-3΄ and 5΄-CGC CAG ACA GCA CTG TGT TG-3΄.

ELISAs, western blots for HIV-1 proteins and *in situ* hybridization for HIV-1 unspliced RNAs were carried out as previously described (13,14). Detection of HIV-1 gp41 used the mouse monoclonal 50–69 (23–27). The reagent was obtained through the NIH AIDS Reagent Program, Division of AIDS, NIAID, NIH: Anti-HIV-1 gp41 monoclonal 50–69 from Dr Susan Zolla-Pazner.

### Plasmids, transfection and RNA interference assays

Plasmids expressing *Bcl-x* reporter minigenes (X2.13 and X2), SRSF9 and SRSF1 were described previously (28–30). Details of plasmids expressing 3XFlag-SRSF10, Flag-SRSF10 and HA-tagged SRSF10 were also previously described (31). Plasmid transfections in HeLa-HIV cells (HeLa rtTA-HIV-ΔMls cells as described in references 13,18,19) or 293 cells (EcR 293 cells from Thermo Fisher Scientific) were carried out with polyethyleneimide (Polysciences Inc.) or Lipofectamine 2000 (Invitrogen) according to the manufacturer's instructions.

The HIV-YFP plasmid is based on phRL-null (Promega) containing the pNL4.3 HIV promoter and the YFP coding sequence in replacement of the RLuc gene. The CMV-Tat plasmid has been described previously (32).

The siRNA used to knockdown the expression of SRSF10, siGENOME SMARTpool-Human SRSF10, was purchased from Dharmacon and transfected into cells at a concentration of 100 nM using Lipofectamine 2000 (Invitrogen). Proteins or RNA were extracted from mock-transfected and siRNA-transfected cells at 72 h post-transfection.

### RNA immunoprecipitation and RT-qPCR analysis

HeLa-HIV cells were transfected with expression plasmid Flag-SRSF10. After 48 hours transfected cells were treated or not with 10 or 20 μM of 1C8 for another 24 h before the cells were harvested. After washing with PBS, the cell pellet was resuspended in RIPA buffer supplemented with protease and RNase inhibitors. Cells were lysed by sonication and the insoluble material was removed by centrifugation at 13 000 × *g* for 10 min at 4°C. The supernatant was precleared by incubation for 1 h at 4°C with SureBeads™ protein G magnetic beads (BioRad) previously blocked with yeast tRNA. An aliquot of the precleared supernatant was removed to be used as the input sample. Precleared lysates of equal protein quantities were incubated overnight at 4°C with SureBeads™ protein G magnetic beads (BioRad) previously coupled for 1 h at room temperature with monoclonal anti-FLAG^®^ M2 antibody (F3165, Sigma-Aldrich). Beads were collected, washed four times with RIPA buffer and resuspended in elution buffer (1% SDS, 5 mM EDTA, 10 mM DTT, 50 mM Tris–HCl pH 7.4). RNA was extracted using TRIzol, resuspended in 15 μl of H_2_O, treated with DNase I for 15 min at 37°C. Two hundred nanogram of RNA measured for integrity was reverse transcribed using random hexamers with Transcriptor reverse transcriptase. Ten nanogram of cDNA were used for quantification in the presence of specific primers (listed in Supplementary Table S4) at 0.2 μM in a 10 μl reaction in triplicates. Fluorescence measurement using SYBR Green was carried out in the ABI 7500 qPCR. To determine the relative abundance of HIV pre-mRNA into the immunoprecipitated complexes, we compared Ct using the input sample (pre-immunoprecipitated) as reference, while the difference between control and 1C8-treated samples was calculated using the 2^−ΔΔ^*^C^*^T^ method and expressed as fold change of HIV-1 pre-mRNA recovered from 1C8-treated samples versus the non-treated control.

### Immunoprecipitation and mass spectrometry analysis

HeLa-HIV cells expressing Flag-SRSF10 and treated or not with various concentrations of 1C8 were cultured in 150 mm plates. Collected cells were washed twice with ice-cold PBS and lysed on ice for 30 min in NET-2 buffer (50 mM Tris–HCl, pH 7.4, 150 mM NaCl, 0.05% (vol/vol) Nonidet P-40 added with EDTA-free protease and phosphatase inhibitors cocktail (Roche Diagnostics GmbH). The lysates were clarified by centrifugation at 13 000 × *g* for 15 min and RNase A solution (0.1 mg/ml of cellular lysate) was added. SureBeads™ protein G magnetic beads (BioRad) were coupled with monoclonal anti-FLAG^®^ M2 antibody (F3165, Sigma-Aldrich) through rotation for 1 hour at room temperature. Equal aliquots of anti-Flag coupled beads were added to pre-cleared Flag-SRSF10-containing cell lysates. After overnight incubation at 4°C, beads were magnetized and washed four times with 0.5 ml of NET2 buffer and four times with 0.5 ml of 20 mM NH_4_HCO_3_. Beads were resuspended in 50 μl of 20 mM NH_4_HCO_3_ buffer containing 1 μg of Trypsin Gold (Promega) and incubated overnight at 37°C while shaking. The reaction was stopped by adding formic acid to 1% final concentration. The supernatant was transferred to a new tube, while beads were resuspended in 50 μl of a solution containing 60% acetonitrile, 0.1% formic acid and incubated for 5 min at room temperature. Both supernatants were pooled and lyophilized using a speedvac. Peptides were resuspended in 30 μl of 0.1% of trifluoroacetic acid and proceeded to desalting using ZipTip C18 (Millipore) as recommended by the manufacturer. Eluted peptides were lyophilized and resuspended in 25 μl of 1% formic acid. Trypsin digested peptides loaded onto an Acclaim PepMap100 C18 column (0.3 mm id × 5 mm, Dionex Corporation) were separated using a Dionex Ultimate 3000 nanoHPLC system. The HPLC system was coupled to an OrbiTrap QExactive mass spectrometer (Thermo Fisher Scientific Inc.) via an EasySpray source. Data acquired using the Xcalibur software were processed, searched and quantified using the MaxQuant software package version 1.4.1.2 (33).

### Transcriptome analysis

Total RNA was purified from HeLa-HIV cells treated with various concentrations of 1C8 in quadruplicate (0, 1, 5, 10 and 20 μM) using the RNAeasy mini kit (Qiagen). A total of 10 μg of RNA was used to generate a TruSeq library following the manufacturer's protocol (Illumina). The library was then sequenced for 51 cycles on an Illumina HiSeq-2500 platform. The resulting 51 nt reads were mapped to the human genome (hg19) with TopHat v2.0.10, using the following parameters: *–no-coverage-search*.

On average for the quadruplicate samples, a total of 19.0, 18.0, 18.8, 18.8 and 19.1 M 51-nt reads were generated for the control, 1, 5, 10 and 20 μM of 1C8, respectively. On average, 96.0%, 96.2%, 96.3%, 96.6% and 96.6% of reads were successfully aligned, and 84.9%, 85.1%, 85.3%, 85.4% and 85.7% of total reads in each respective library were uniquely mapped to the human genome. TPM (transcripts per million mapped reads) were then calculated for each gene by counting the number of uniquely mappable reads that fell within the exonic region for all annotated genes in the Ensembl release 73. The counts for each gene were normalized using the total length of their respective exonic regions. For the MISO analysis, all replicates for a given treatment were merged into a single file.

## RESULTS

### 1C8 affects HIV-1 transcription and splicing

**Figure 1.**
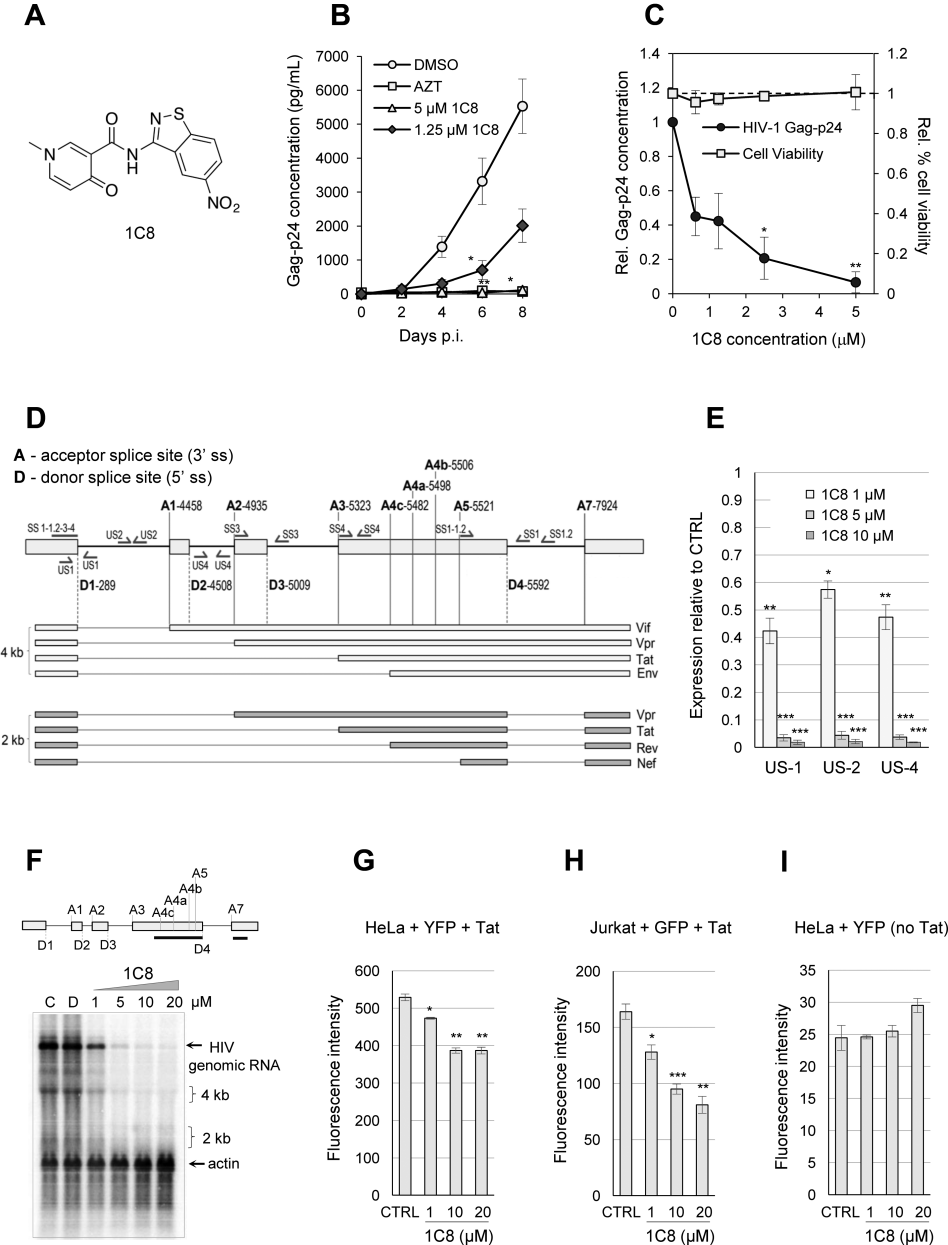
Effect of 1C8 on HIV-1 expression. (**A**) Chemical structure of 1C8. (**B**) Effect of 1C8 on HIV-1 replication in PBMCs. Assay monitoring HIV-1 BaL virus replication over a period of eight days post-infection (p.i.) as measured by Gag-p24 antigen by ELISA (*n* > 3, 3–4 donors). PBMCs were infected with HIV-1 BaL (MOI < 0.01) and treated on days 0 and 4 post-infection with DMSO, AZT (3.74 μM), or 1C8 at the concentrations indicated. Error bars indicate standard error of the mean (SEM) of replicate wells from an independent experiment. (**C**) Effect of increasing concentrations of 1C8 on cell viability in PBMCs. Culture supernatants were measured for Gag-p24 antigen by ELISA and expressed relative to DMSO treatment (*n* ≥ 3, *P ≤ 0.05, **P ≤ 0.01 and ***P ≤ 0.001). The effect of the compounds on cell viability was measured by trypan blue exclusion as a percentage of total cells and expressed relative to percent cell viability with DMSO treatment. (**D**) Map of donor and acceptor splice sites (5΄ss and 3΄ss, respectively) used for the production of the major HIV mRNAs. The main viral proteins produced from each mRNA are indicated. (**E**) Quantitative RT-PCR assays from HeLa-HIV cells treated with 1C8. Histograms depicting the impact of 1C8 on amplicons derived from unspliced segments of HIV-1 transcripts. The intensity of products was normalized relative to the amount produced in the controls (which corresponds to an arbitrary unit value of 1). (**F**) Northern analysis of HIV-1 transcripts following treatment with various concentrations of 1C8 for 24 h. The HIV probe is indicated by a black line below the HIV map. A β-actin probe was used to reveal loading variations. Positions of 4 and 2 kb species are indicated. (**G**) HeLa cells were co-transfected with a HIV-YFP plasmid and a CMV-Tat plasmid in the presence of the indicated concentrations of 1C8. Total YFP fluorescence of YFP positive cells was measured 36 h post-transfection. (**H**) Jurkat cells were co-transfected with a HIV-YFP plasmid and a CMV-Tat plasmid in the presence of the indicated concentrations of 1C8. Total YFP fluorescence of YFP positive cells was measured 36 h post-transfection. (**I**) HeLa cells were transfected with the HIV-YFP plasmid in the presence of the indicated concentrations of 1C8. Total YFP fluorescence of YFP positive cells was measured 36 h post-transfection. In all cases, asterisks represent significant *P* values (two-tailed Student's *t* test) comparing the means between 1C8-treated samples and their respective controls; *P < 0.05, **P < 0.01 and ***P < 0.001.

Based on the structure of IDC16 (16), a diversity-driven library synthesis-screening approach was used to design alternative mimics of IDC16 with anti-HIV activity. Using the T cell-based CEM-GXR reporter cell line, 1C8 was the most active compound (Figure 1A). With half maximal effective concentration (EC_50_) values ranging between 0.6 and 1.5 μM, 1C8 inhibited replication of wild-type HIV-1 (subtypes A and B), as well as representative strains resistant to drugs that target viral reverse transcriptase, protease, integrase and the cellular co-receptor CCR-5 (17). Moreover, 1C8 elicited very modest changes in cell viability (17). Here, we now show that 1C8 also inhibits HIV-1 replication in human peripheral blood mononuclear cells (PBMCs) (Figure 1B), with no significant impact on cell viability (Figure 1C).

To determine the impact of 1C8 on HIV-1 transcription and splicing, we used a human HeLa cell line stably transduced with a modified X4 HIV-1 (LAI) provirus regulated by a Tet-ON system (HeLa rtTA-HIV-ΔMls or HeLa-HIV) (13,34,35). This provirus contains mutations that abrogate the function of the TAR RNA element and the Tat gene, thus rendering HIV-1 expression strictly dependent on the Tet-ON system. The effects of 1C8 on HIV-1 gene expression were monitored by initiating treatment of HeLa-HIV cells with 1C8 one hour prior inducing viral expression with doxycycline for 24 h. We performed quantitative RT-PCR assays using three different sets of primers located in introns or flanking an exon/intron junction to monitor HIV-1 genomic expression (US1, US2 and US4 pairs of primers, Figure 1D). The results indicate that 1 μM of 1C8 elicits a 40–60% decrease in HIV-1 RNA accumulation, with <2% of transcripts remaining when 10 μM of 1C8 is used (Figure 1E). A Northern analysis confirmed the impact of 1C8 on gene expression, with reductions in viral mRNAs that are consistent with our qRT-PCR analysis (Figure 1F).

To test the impact of 1C8 on HIV-1 expression when driven by the natural HIV-1 promoter, we transfected HIV-YFP and CMV-Tat plasmids in HeLa cells. In these conditions, the effect of 1C8 on HIV-1 expression was smaller than in HeLa-HIV cells, with 1 and 10 μM of 1C8, respectively promoting an average drop of 11% and 27% in expression (Figure 1G). Transfection of the same plasmids in Jurkat cells also indicated moderate drops in YFP expression at 1 μM and 10 μM of 1C8 (∼22% and 42%, respectively) (Figure 1H). Monitoring YFP expression in HeLa cells in the absence of Tat indicated that basal HIV-1 expression was not affected by 1C8 (Figure 1I). We also tested the impact of 1C8 on the expression of HIV-1 when programmed by the natural endogenous HIV-1 promoter in the CD4+ T cell line 24ST1NLESG (18,36). Ten μM of 1C8 promoted a 60% drop in the accumulation of unspliced HIV-1 mRNAs (Supplementary Figure S1), an effect that is considerably less than on the Tet-ON HIV-1 promoter. Thus, although 1C8 strongly affects expression from the Tet-ON HIV-1 promoter, Tat-dependent expression from the natural HIV-1 promoter is more weakly affected by 1C8.

**Figure 2.**
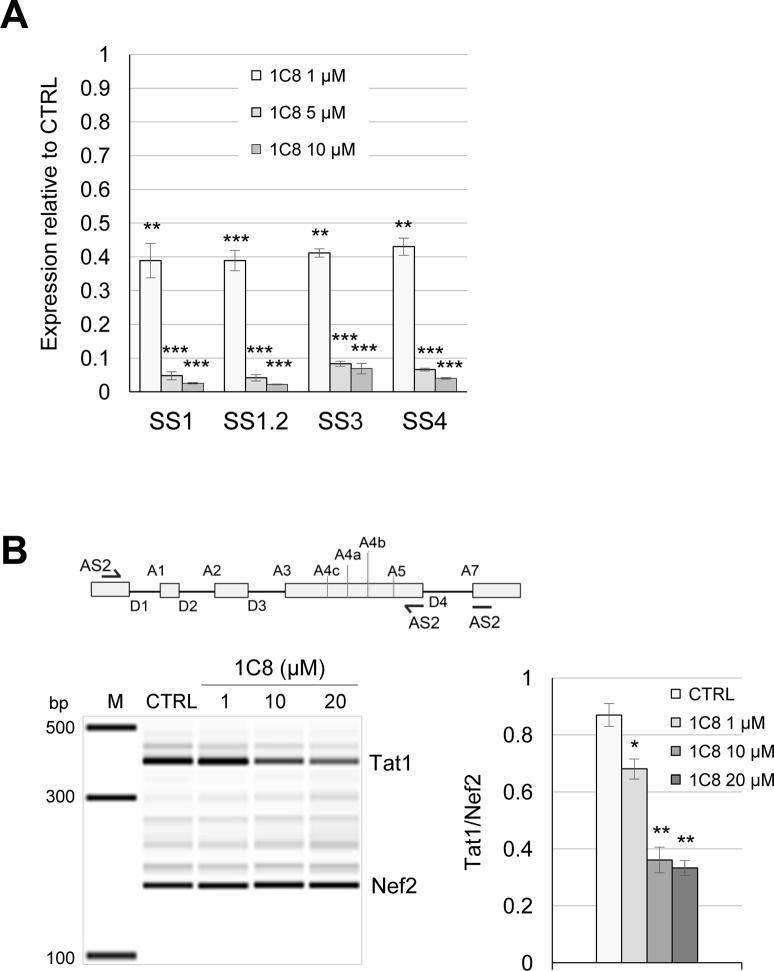
Effect of 1C8 on HIV-1 splicing. (**A**) Quantitative RT-PCR assays from HeLa-HIV cells treated with 1C8. Histograms depicting the impact of 1C8 on selected amplicons produced with the primers indicated in Figure 1A. The intensity of products was normalized relative to the amount produced in the controls (which corresponds to an arbitrary unit value of 1). (**B**) Map of donor and acceptor splice sites (5΄ss and 3΄ss, respectively) used for the production of the major HIV-1 mRNAs. The AS2 set of primers was used to monitor the production of Tat1 and Nef2. Endpoint RT-PCR assay from HeLa-HIV cells treated with 1C8. Representative electropherograms showing the Tat1 (D1/A3) and Nef2 (D1/A5) amplicons. Histograms depicting the impact of 1C8 on Tat1 and Nef2 production. In both panels, asterisks represent significant *P* values (two-tailed Student's *t* test) comparing the means between 1C8-treated samples and their respective controls; *P < 0.05, **P < 0.01 and ***P < 0.001.

**Figure 3.**
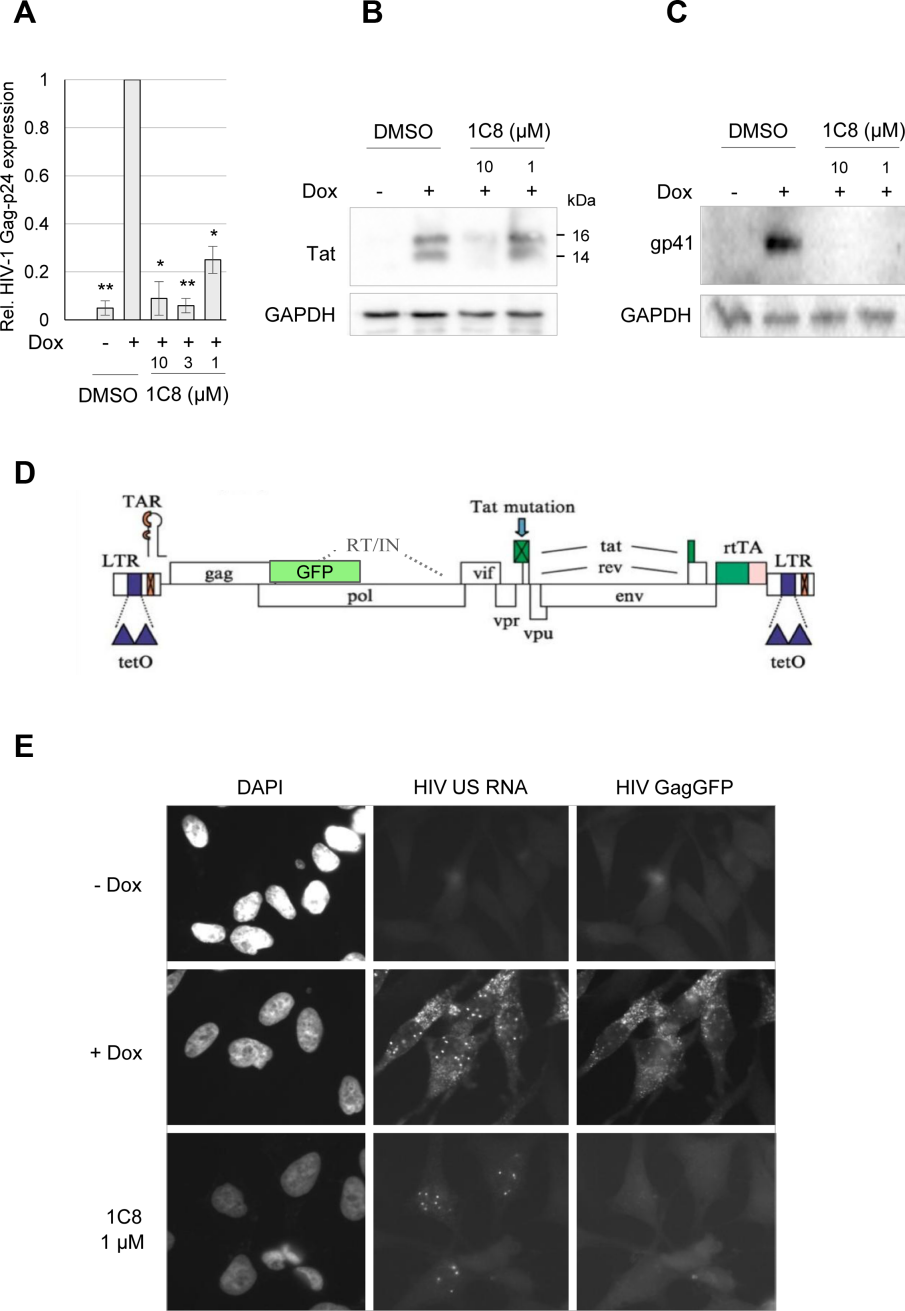
Impact of 1C8 on HIV-1 protein expression and localization of unspliced viral pre-mRNA. HeLa-HIV cells were treated with either DMSO or 1C8 at the indicated doses for 4 h prior to addition of doxycycline (Dox) to induce provirus expression. Twenty-four hours after Dox addition, cells were harvested and viral protein levels were assessed by ELISA for HIV Gag-p24 (**A**) or immunoblots for viral proteins Tat (**B**) and Env-gp41 (**C**). In panel A, asterisks represent significant *P* values (two-tailed Student's *t* test) comparing the means between 1C8-treated samples and their respective controls; *P < 0.05, **P < 0.01 and ***P < 0.001. (**D**) Structure of the HIV-1 provirus used to generate the HeLa cell line with the GagGFP fusion. The provirus has elements (TetO sites, rtTA) required for regulation by doxycycline (Dox). (**E**) Effect of 1C8 on HIV-1 unspliced (US) RNA localization. Cells were incubated in the absence (–) or the presence (+) of Dox for 24 h, then fixed, stained with DAPI and imaged using a Leica DMR epifluorescent microscope. HeLa HIV GagGFP cells were treated with either DMSO or 1C8 (1 μM) for 4 h prior to addition of Dox to induce provirus expression. Twenty-four hours after Dox addition, cells were fixed and HIV-1 US RNA localization was determined by *in situ* hybridization. Magnification 600×.

Since 1C8 is derived from a compound that was designed to target splicing (16), we asked if 1C8 also affected HIV-1 RNA splicing. HIV-1 RNA splicing was analyzed by quantitative RT-PCR using different combinations of forward and reverse primers (Figure 1D). To monitor the removal of specific introns, we used forward primers that cover the junction of spliced exons (SS1, SS1.2, SS3 and SS4; Figure 1D). The levels of amplified products derived from D1/A5 splicing (Nef, using the SS1 and SS1.2 pairs of primers), D1/A2 splicing (Vpr1, using the SS3 pair of primers) and D1/A3 splicing (Tat1, using the SS4 pair of primers) were reduced on average by ∼60% with 1 μM of 1C8 (Figure 2A). As this drop matches the decrease in expression imposed by 1C8, either 1C8 has no impact on these HIV-1 splicing events, or small changes are obscured by the general drop in HIV expression. To circumvent this problem, we opted for monitoring several spliced viral RNAs simultaneously and determine if 1C8 changed their relative abundance. Endpoint RT-PCR with primers AS2 allows to amplify products derived from several HIV transcripts, with Tat1 and Nef2 being the most abundant products detected (Figure 2B). Notably, 1C8 reduced the production of Tat1 (through D1/A3 and D4/A7 splicing) relative to Nef2 (through D1/A5 and D4/A7 splicing) (Figure 2B), indicating an impact on the production of splice variants. Thus, the combined effect of 1C8 on HIV-1 transcription and splice site selection may lead to critical imbalances in the production of components required for viral replication. Consistent with this view, 1C8 has a negative impact on the production of viral proteins (Tat, Gag-p24 and Env protein gp41), as determined by ELISA and immunoblot analyses (Figure 3A–C). Monitoring unspliced viral pre-mRNA by fluorescent *in situ* hybridization indicates that while unspliced HIV-1 transcripts are detected in the nucleus using 1 μM of 1C8, their cytoplasmic levels were strikingly reduced (Figure 3D and E). Defects in cytoplasmic transport of unspliced HIV-1 transcripts would be consistent with a defect in Rev, due to either loss of expression or inhibition of function. Although our RT-PCR analysis did not detect 1C8-mediated alterations in splice variants encoding Rev (data not shown), we cannot rule out that Rev protein expression or localization is affected.

### 1C8 affects the activity of SRSF10

**Figure 4.**
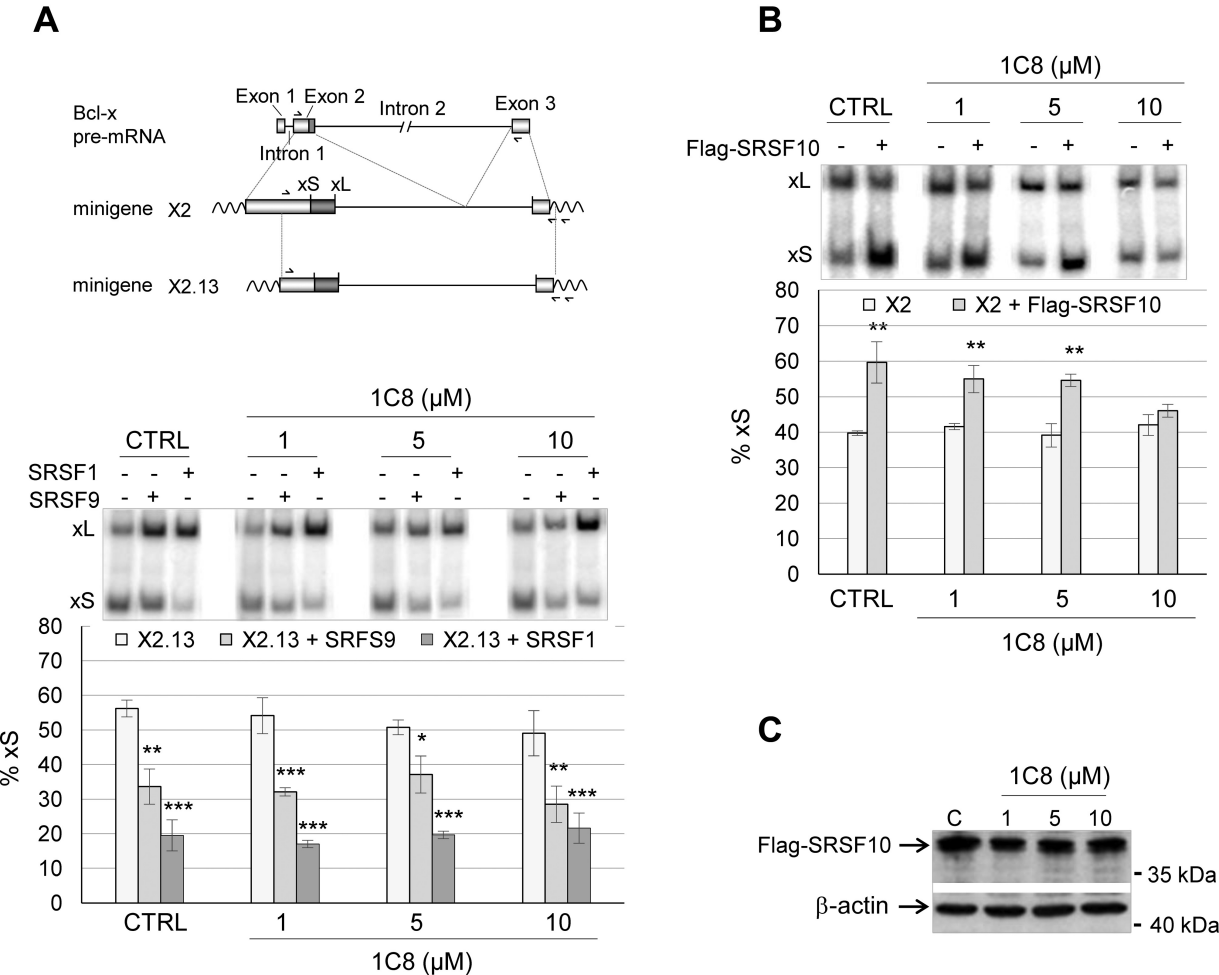
Impact of 1C8 on the activity of SRSF1, SRSF9 and SRSF10. (**A**) 293 cells were co-transfected with a plasmid carrying *Bcl-x* minigene X2.13 and a plasmid containing the CMV promoter driving the expression of the SRSF1 or SRSF9 cDNA, as described previously in Cloutier *et al.* (30). The assay was performed in the presence of the indicated concentrations of 1C8. A map of the *Bcl-x* gene is shown on top to illustrate portions used to create minigenes X2.13 and X2 (see panel B). The position of the 5΄ splice of Bcl-xS (xS) and Bcl-xL (xL) is shown as well as the position of primers used in the RT-PCR assays. (**B**) 293 cells were co-transfected with the *Bcl-x* minigene X2 and a Flag-SRSF10 plasmid in the presence of the indicated concentrations of 1C8. The percentage of Bcl-xS over the sum of both Bcl-xS and Bcl-xL is shown in histograms. (**C**) Immunoblot performed to monitor the expression of Flag-SRSF10. In all cases, asterisks represent significant *P* values (two-tailed Student's *t* test) when comparing the means between samples and their respective controls; *P < 0.05, **P < 0.01 and ***P < 0.001.

Since 1C8 affects splice site selection, and is derived from a molecule that affects the activity of SRSF1, we tested whether 1C8 alters SRSF1 function. Note that another molecule, ABX-464, whose development was also inspired by the structure of IDC16, stimulates HIV-1 mRNA splicing and interacts with the cap binding complex (37). For this assay, we used *Bcl-x* reporter minigenes. *Bcl-x* is alternatively spliced through the use of two 5΄ splice sites (Figure 4A, top) to produce the Bcl-xL and Bcl-xS splice variants. SRSF1 is a regulator of *Bcl-x* splicing that increases the production of Bcl-xL (30,38,39). Plasmids expressing SRSF1 or the related protein SRSF9, which also favors the production of Bcl-xL (30), were co-transfected into 293 cells with the *Bcl-x* reporter minigene X2.13. In agreement with previous results, both SRSF1 and SRSF9 reduced the production of Bcl-xS (Figure 4A). The addition of 1C8 at concentrations of up to 20 μM did not alter the splice site shifting activity of either SRSF1 or SRSF9 (Figure 4A). We then tested the impact of 1C8 on the activity of another SR protein, SRSF10, that when overexpressed, stimulates the production of Bcl-xS on transcripts derived from *Bcl-x* minigene X2 (Figure 4B) (31). In this case, 10 μM of 1C8 completely abrogated the SRSF10-induced splicing shift (Figure 4B). Since 1C8 has little effect on the expression level of the Flag-SRSF10 protein (Figure 4C), we conclude that 1C8 affects the splicing regulatory function of SRSF10.

### SRSF10 controls HIV-1 splicing

**Figure 5.**
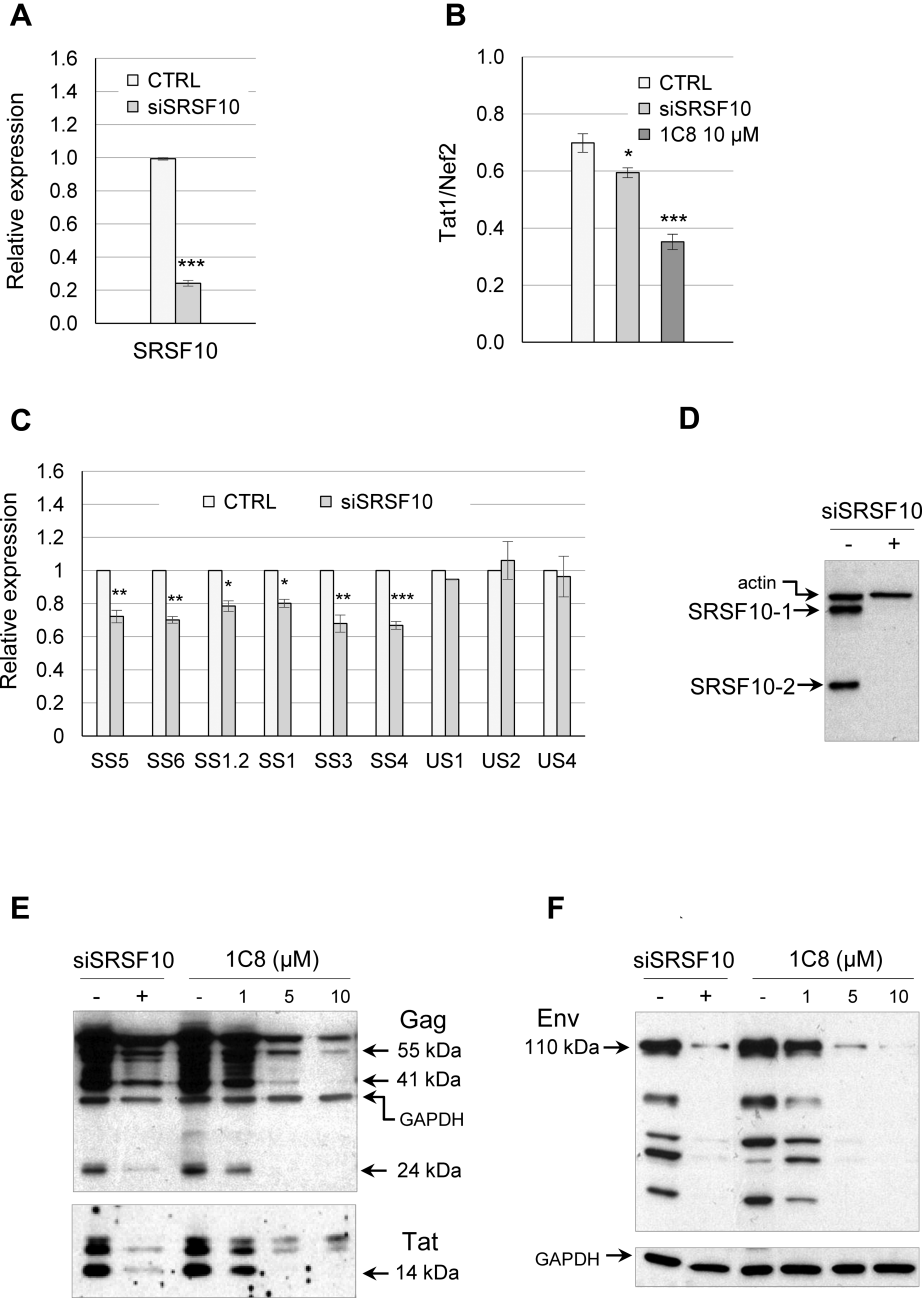
Impact of the depletion of SRSF10 on HIV-1 expression. (**A**) Quantitative RT-PCR was used to assess the level of depletion of endogenous SRSF10 in the HeLa-HIV cell line. (**B**) The AS2 set of primers was used to monitor the production of Tat1 and Nef2. Endpoint RT-PCR assay from HeLa-HIV cells depleted of SRSF10 by siRNA or treated with 10 μM of 1C8. The histograms show the impact of the depletion and of 1C8 on the ratio of Tat1 and Nef2 products. (**C**) Using primer pairs described in Figure 1D, quantitative RT-PCR assays were performed on RNA from HeLa-HIV cells treated with siSRSF10. The intensity of products in histograms was normalized relative to the amount produced in the controls (which corresponds to an arbitrary unit value of 1). Asterisks indicate significant *P* values when comparing the difference between the means of values from samples treated with siSRSF10 versus values from mock-treated samples; *P < 0.05, **P < 0.01 and ***P < 0.001. (**D**) Knockdown of SRSF10 with siSRSF10 as verified by immunoblotting. (**E**) Western analysis of HIV-1 Gag, Tat and Env proteins. Equivalent loading was confirmed by reprobing blots with anti-GAPDH antibody.

If 1C8 impacts HIV-1 splicing by affecting the activity of SRSF10, then HIV-1 splicing should be sensitive to a reduction in the level of SRSF10. To test this prediction, we used RNA interference to deplete SRSF10 in the HeLa-HIV cell line (Figure 5A). Endpoint RT-PCR analysis using the AS2 pair of primers (Figure 2B) indicated that the partial siRNA-mediated depletion of SRSF10 affected splice site selection in the same manner as 10 μM of 1C8, compromising the production of Tat1 relative to Nef2 (Figure 5B). Quantitative RT-PCR analysis indicated that a partial depletion of SRSF10 reduced by 20–30% the level of products derived from the D4/A7 splice (SS5 and SS6 pairs of primers), the D1/A5 splice (SS1 and SS1.2 pairs of primers), the D1/A2 splice (SS3 pair of primers) and the D1/A4 splice (SS4 pair of primers). The siRNA against SRSF10 had no impact on HIV Tet-ON expression based on RT-PCR products derived from intron-containing transcripts (US1, US2 and US4 pairs of primers) (Figure 5C). These results suggest that SRSF10 contributes to HIV-1 RNA splicing regulation, and that the impact of 1C8 on HIV-1 splicing may be caused, at least in part, by altering SRSF10 activity.

To interrogate further how SRSF10 impacts HIV-1 expression, we tested the impact of the siRNA-mediated depletion of SRSF10 on the production of late viral proteins in HeLa-HIV cells. Clearly, the depletion of SRSF10 (Figure 5D) reduced the steady-state levels of Gag, Tat and Env proteins in a manner that was similar to the drop obtained by 5 μM of 1C8 (Figure 5E and F). Although a slight drop in Tat protein was also seen at 1 μM of 1C8, this reduction was not seen in the assay presented in Figure 3B.

### 1C8 dephosphorylates SRSF10

**Figure 6.**
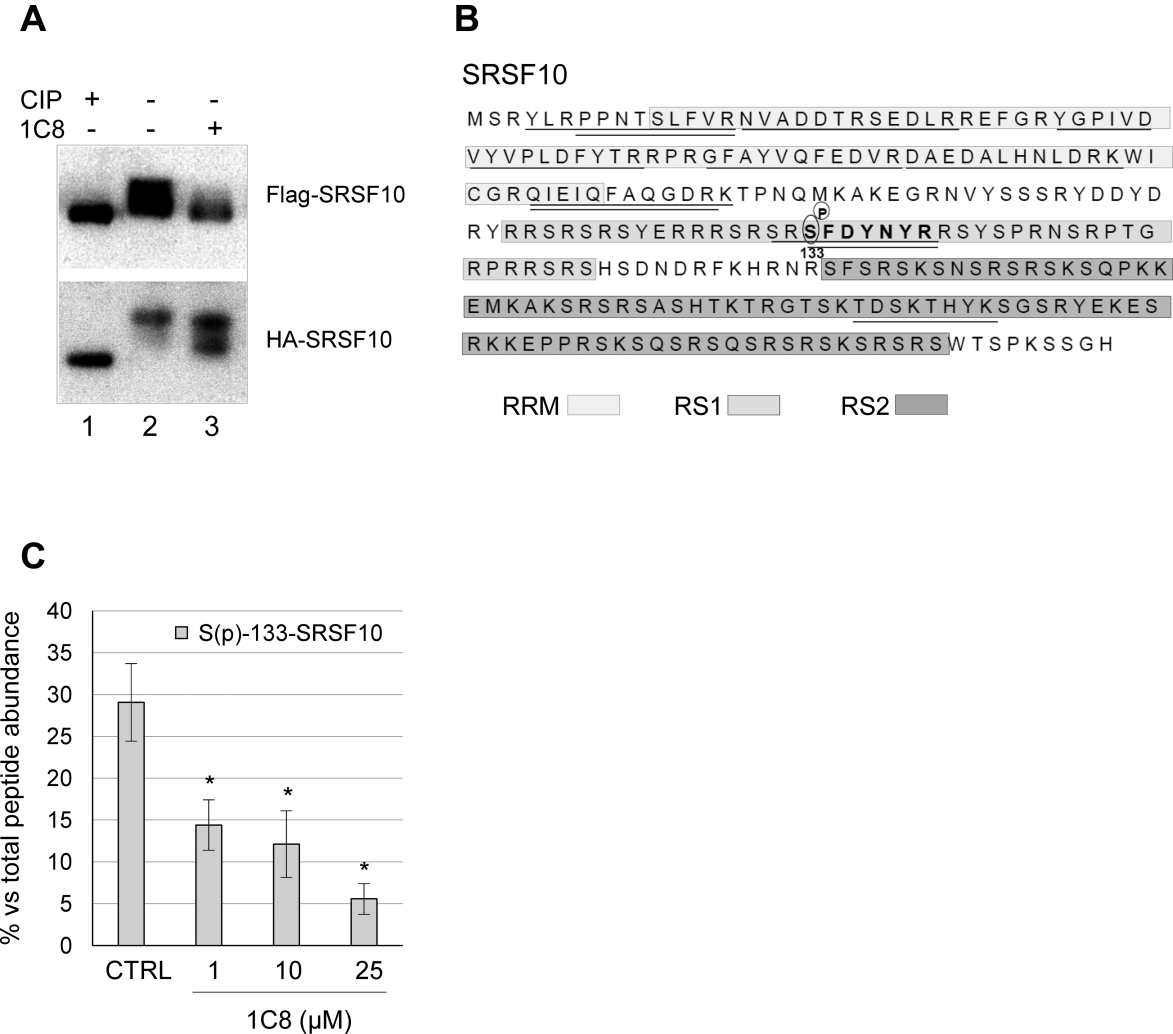
1C8 affects the phosphorylation of SRSF10. (**A**) Total cellular extracts of HeLa-HIV cells transfected with Flag-SRSF10 or HA-SRSF10 expression plasmids were prepared 24 h after treatment or not with 20 μM of 1C8. Aliquots of extract from untreated cells were incubated with or without calf intestinal phosphatase (CIP) for 15 min at 37°C. Extracts were fractionated on a denaturing gel and proteins were transferred on nitrocellulose to reveal tagged SRSF10. (**B**) Amino acid sequence of the SRSF10-1 protein showing the different domains (RRM, RS1 and RS2) in shaded boxes. The SRSF10 peptides identified by LC–MS/MS analysis after immunoprecipitation of cellular extracts with the anti-Flag antibody are underlined, while the SFDYNYR peptide which carries a phosphorylated serine at position 133 is shown in bold. (**C**) The percentage of phosphorylated SFDYNYR relative to the non-phosphorylated version recovered from HeLa-HIV cells treated with the indicated doses of 1C8 is plotted. Asterisks represent *P* values (two-tailed Student's *t* test) comparing the means between 1C8-treated samples and the control; *P < 0.05, **P < 0.01 and ***P < 0.001.

SRSF10 is a phosphorylated protein, and its dephosphorylation can convert SRSF10 from a positive regulator into a repressor (40,41). Moreover, we have recently associated changes in alternative splicing with the dephosphorylation of SRSF10 (31). Thus, we asked whether 1C8 altered the phosphorylation status of SRSF10. Treating extracts with calf intestinal phosphatase (CIP) produces a dephosphorylated version of SRSF10 with faster mobility than phosphorylated forms in gel conditions that maximize resolution (31,42). We reproduced this observation when extracts from HeLa-HIV cells expressing Flag-SRSF10 and HA-SRSF10 proteins were treated with CIP (Figure 6A). Using this system, we observed that treating cells with 20 μM of 1C8 for 24 h converted SRSF10 into faster-migrating forms that suggested partial dephosphorylation (Figure 6A). To identify the changes in phosphorylation elicited by 1C8, we used an anti-Flag antibody to recover Flag-SRSF10 from extracts of HeLa-HIV cells treated with various concentrations of 1C8. This assay was performed in duplicate and the recovered proteins were analyzed by LC/MS-MS. Among the 12 peptides that uniquely matched SRSF10, peptide SFDYNYR carries a serine at position 133 that was phosphorylated in 30% of the peptide recovered (Figure 6B and C). The recovery of this phosphorylated peptide relative to the non-phosphorylated version was decreased 2- to 6-fold in extracts from cells treated with 1–25 μM of 1C8 (Figure 6C). Thus, 1C8 promotes the dephosphorylation of Flag-SRSF10 at serine 133. Although we did not identify other phosphorylated peptides matching SRSF10, other dephosphorylation events not detected by our partial peptide coverage (30%) are likely to have occurred based on the change in the SRSF10 gel migration profile from 1C8-treated cells (Figure 6A). Notably, removing serine 133 from Flag-SRSF10 compromises the activity of SRSF10 and the dual removal of Ser131 and Ser133 alters the interaction of SRSF10 with other splicing factors (31).

### 1C8 alters the interaction of SRSF10 with HIV-1 transcripts and with splicing factors

**Figure 7.**
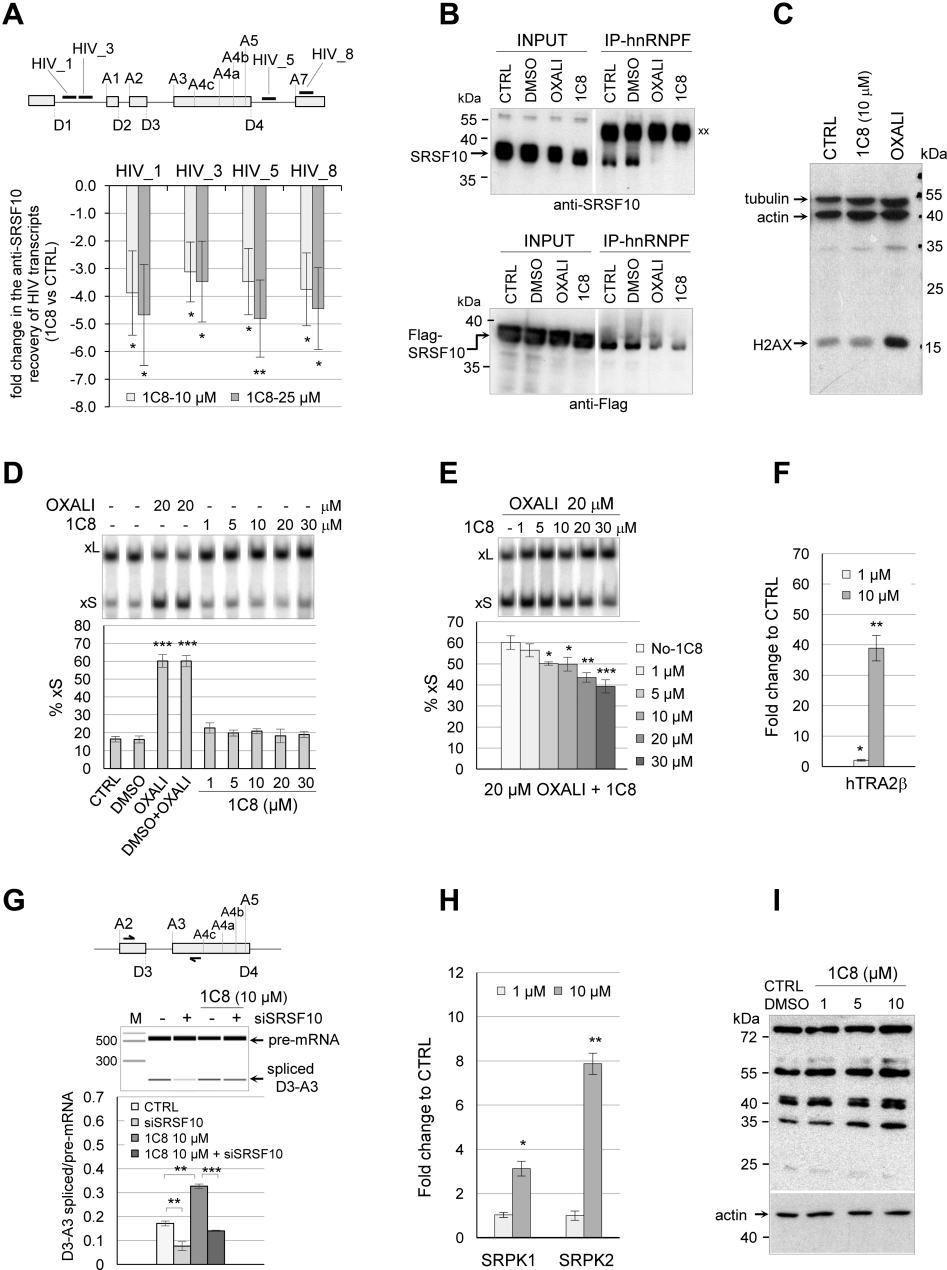
1C8 affects the interaction of SRSF10 with HIV-1 transcripts and splicing factors. (**A**) The RNA recovered from anti-Flag immunoprecipation of extracts from HeLa-HIV cells treated or not with 1C8 was quantitated by RT-PCR using primers mapping to different portions of the HIV-1 pre-mRNA. The approximate position of amplicons on the HIV-1 genome is shown on top (HIV_1, HIV_3, HIV_5 and HIV_8). For each amplicon, the fold change between values in the 10 or 20 μM samples of 1C8 versus the non-treated control is plotted. (**B**) Immunoprecipitation of SRSF10 (top panel) and 3XFlag-SRSF10 (bottom panel) was performed with anti-hnRNP F antibodies using extracts prepared from non-treated 293 cells (CTRL) or treated with DMSO, 20 μM oxaliplatin, or 20 μM 1C8. The input content of SRSF10 and 3XFlag-SRSF10 in various samples is shown and represents 1/50th of the samples used for the immunoprecipitation. Immunoprecipitates were fractionated on gel and proteins were transferred to nitrocellulose that were decorated with anti-SRSF10 and anti-Flag antibodies. ‘xx’ indicates the large immunoglobulin subunit used for the immunoprecipitation that reacts with the secondary antibody. (**C**) Total proteins from control 293 cells and cells treated for 24 h with 20 μM 1C8 or 20 μM of oxaliplatin were fractionated by SDS-PAGE gel and transferred to nitrocellulose that was decorated with antibodies against phosphorylated H2AX, β-actin and tubulin. (**D**) The impact of 1C8 on *Bcl-x* splicing was tested by treatment of 293 cells for 24 h with the indicated concentrations of 1C8 and oxaliplatin. Total RNA was extracted and the *Bcl-x* splicing profile was revealed by RT-PCR. (**E**) The indicated concentrations of 1C8 were added to 293 cells one hour before adding 20 μM of oxaliplatin. After 24 h, total RNA was extracted and the percentage of Bcl-xS mRNA splice variant was determined. (**F**) The recovery of hTRA2β peptides from the anti-Flag immunoprecipitation of ribonuclease-treated samples was quantitated by LC–MS/MS analysis after normalization for equivalent of SRSF10 in all recovered samples. The presence of the hTRA2β peptides in the 1C8-treated samples was compared with control mock-treated samples. The mean fold change of recovered hTRA2β peptides is shown. (**G**) Impact of 1C8 and siSRSF10 on HIV-1 D3/A3 splicing in HeLa-HIV cells. Primer pairs indicated on top were used to perform endpoint RT-PCR, allowing to amplify both unspliced and spliced products. Analysis and visualization of amplified products were done using the LabChip HT DNA assay on a Caliper LC-90 automated microfluidic station. Typical electropherograms are shown, as well as histograms from an assay done in triplicate.(**H**) Similar to panel F, the mean fold change of SRPK1 and SRPK2 peptides recovered from 1C8-treated and mock-treated samples is shown in histograms. (**I**) Immunoblot with mAb104, which recognizes a phosphoepitope shared by the major SR proteins (51). The blot used gel-fractionated protein samples from HeLa cells treated for 18 h with the indicated concentrations of 1C8. In panels A and D–H, asterisks represent *P* values (two-tailed Student's *t* test) comparing the means between samples and their respective controls; *P < 0.05, **P < 0.01 and ***P < 0.001.

To understand how 1C8 affects the function of SRSF10, we first asked if 1C8 changed the association of SRSF10 with HIV-1 transcripts. We used quantitative RT-PCR to measure the amount of HIV-1 RNA recovered by immunoprecipitation with the anti-Flag antibody using HeLa-HIV cells expressing Flag-SRSF10. qRT-PCR was performed with four sets of primers that mapped in regions located in Gag and Env reading frames, as well as the last HIV-1 exon (Figure 7A). The recovered material was treated with DNase I to eliminate a potential contribution of contaminating genomic DNA. As shown in Figure 7A, this analysis reveals that 10 and 25 μM of 1C8 decreased by 3- to 4.5-fold the association of SRSF10 with HIV-1 transcripts.

SRSF10 interacts with hnRNP F/H but this interaction is disrupted when cells are treated with oxaliplatin which dephosphorylates SRSF10 (31). We tested if 1C8 similarly impacts the SRSF10/hnRNP F interaction. First, we observed that 1C8 does not affect the expression of hnRNP F (Supplementary Figure S2). Second, an immunoprecipitation assay performed with anti-hnRNP F antibodies revealed that 1C8 disrupts the interaction of hnRNP F with both endogenous SRSF10 and with Flag-SRSF10 in a manner that matches the impact of oxaliplatin (Figure 7B). Although both oxaliplatin and 1C8 promote the dephosphorylation of S133 on SRSF10 (31), other events specific to each compound may differentially impact SRSF10 activity and splicing. For example, oxaliplatin, but not 1C8, elicits DNA damage, as monitored by the phosphorylation of H2AX (Figure 7C). Moreover, and in contrast to oxaliplatin, 1C8 does not shift *Bcl-x* splicing when SRSF10 is not overexpressed (Figure 4B for a *Bcl-x* minigene and Figure 7D for endogenous *Bcl-x*). Notably, 1C8 antagonizes the *Bcl-x* splicing shift induced by oxaliplatin (Figure 7E). Thus, while oxaliplatin co-opts SRSF10 to alter *Bcl-x* splicing (31), the impact of 1C8 on *Bcl-x* splicing is more consistent with an inactivation of SRSF10. Compound-specific post-translational modifications not identified in our mass spectrometry analysis of SRSF10, or an impact on the activity of other *Bcl-x* splicing regulatory factors, may contribute to these distinctive outcomes.

Phosphorylated SRSF10 was shown previously to interact with hTRA2α, and this interaction is lost when SRSF10 is fully dephosphorylated (41). Given that the related protein hTRA2β regulates HIV-1 splicing (14,43), we tested if Flag-SRSF10 interacts with hTRA2β, and if so, whether this interaction is altered by 1C8. We interrogated our mass spectrometry peptide data from the anti-Flag immunoprecipitation of ribonuclease-treated samples for the presence of hTRA2β peptides. Several hTRA2β peptides were recovered that were not found in mock immunoprecipitated samples, indicating that SRSF10 interacts with hTRA2β. Surprisingly, for equivalent amounts of SRSF10 in each sample, the level of hTRA2β peptides increased nearly 40-fold in samples from cells treated with 10 μM of 1C8 (Figure 7F). hTRA2β regulates HIV-1 splicing specifically by binding to an exonic splicing enhancer that stimulates splice site D3 (43). 1C8 stimulated the production of D3/A3 spliced product, whereas the depletion of SRSF10 repressed it (Figure 7G). Moreover, the depletion of SRSF10 compromised the stimulation of D3/A3 splicing by 1C8, indicating that SRSF10 is required for the impact of 1C8 on D3/A3 splicing. Given that 1C8 does not affect the expression level of hTRA2β (Supplementary Figure S2), our results suggest that 1C8 may stimulate D3/A3 splicing by encouraging the assembly of a SRSF10/hTRA2β enhancer complex.

Another set of relevant SRSF10 interactors include the SR protein kinases SRPK1 and SRPK2, whose recovery was respectively increased 3- and 8-fold by 10 μM of 1C8 (Figure 7H). Given that 1C8 promotes the dephosphorylation of SRSF10, its increased interaction with SR protein kinases may indicate that 1C8 inhibits the activity of these kinases when acting on SRSF10. 1C8 may affect predominantly SRSF10 since the phosphorylation status of the major SR proteins which are substrates for SRPKs was not strongly affected by 1C8 (Figure 7I).

### Impact of 1C8 on cellular gene expression and pre-mRNA splicing

**Figure 8.**
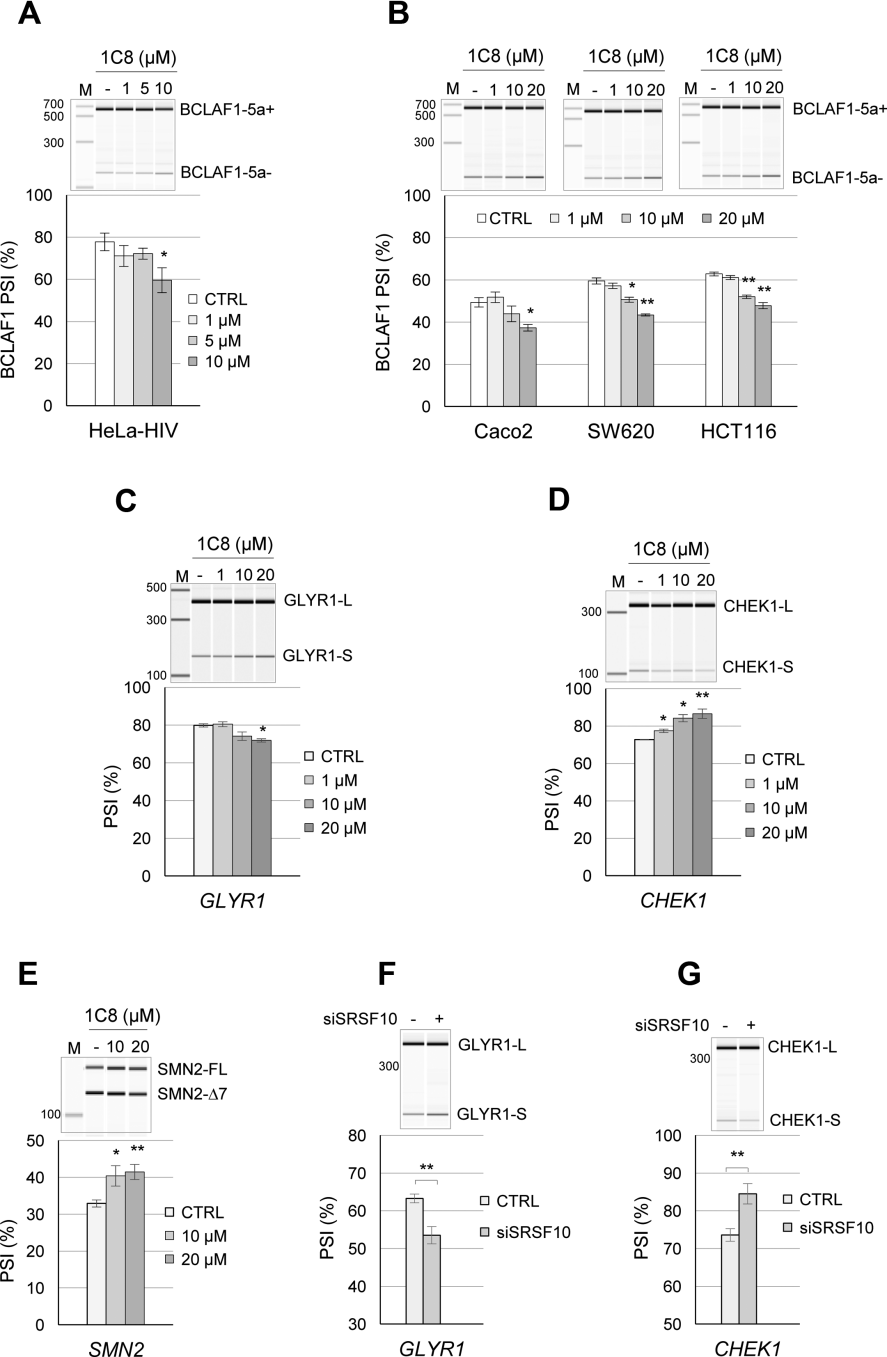
1C8 and the alternative splicing of SRSF10 and hTra2β target transcripts**. (A**) HeLa-HIV cells and (**B**) Caco2, SW620 and HCT116 cells were treated with the indicated concentrations of 1C8. After 24 h, total RNA was extracted and *BCLAF1* splicing products were determined by endpoint RT-PCR. Analysis and visualization of amplified products were done using the LabChip HT DNA assay on a Caliper LC-90 automated microfluidic station. Electropherograms and mean of percent splicing index (PSI) values are shown. (**C** and **D**) HeLa-HIV cells and (**E**) Sma77 cells derived from a SMA patient were treated with the indicated concentrations of 1C8. The splicing products of *GLYR* and *CHEK1* in HeLa-HIV cells and *SMN2* in Sma77 cells were quantitated as described above and the means of percent splicing index (PSI) values are shown, as well as representative electropherograms. (**F** and **G**) Cells were also treated with siSRSF10 and the impact of this treatment on the alternative splicing of the indicated transcripts was monitored.

A recent study carried out in human colon cancer cell lines identified *BCLAF1* as a transcript whose alternative splicing is regulated by SRSF10 (44). The ability of 1C8 to impact the alternative splicing of *BCLAF1* was tested in the HeLa-HIV cell line as well as in three colon cancer cell lines (Caco2, SW620 and HCT116). In all cases, a concentration of 10 μM of 1C8 was required to elicit a >10 percentage points decrease in the production of the exon 5a-containing variant of *BCLAF1* (Figure 8A and B), consistent with the predicted effect of a decrease in the activity of SRSF10. As 1C8 increases the interaction of SRSF10 with hTRA2β, we also tested whether known hTRA2β-controlled splicing events were affected by 1C8. The depletion of hTRA2β promotes exon skipping in *GLYR1, CHEK1* and *SMN2* (45,46). Notably, 1C8 induced exon skipping in *GLYR1* but increased inclusion of *CHEK1* exon 3 in HeLa-HIV cells and *SMN2* exon 7 in the SMA cell line (Figure 8C–E). Knocking down SRSF10 in HeLa-HIV cells shifted splicing of *GLYR1* and *CHEK1* in the same direction as 1C8 (Figure 8F and G). While these results are consistent with the notion that 1C8 affects hTRA2β activity, the different behavior of various units may reflect exon-specific configurations of regulatory splicing elements. For example, if hTRA2β and SRSF10 positively regulate *GLYR1* exon inclusion through distinct elements, 1C8 may promote exon skipping by provoking the dissociation of SRSF10 from the pre-mRNA, as it did on HIV-1 transcripts monitored in Figure 7A. Alternatively, stimulating the formation of a SRSF10/hTRA2β complex in this exon may be inhibitory. In contrast, for *SMN2* and *CHEK1*, 1C8, hTRA2β and the depletion of SRSF10 all stimulated exon inclusion. In these cases, if SRSF10 is normally a negative regulator, by antagonizing hTRA2β binding because of overlapping binding sites, then 1C8, by promoting the dissociation of SRSF10, may stimulate hTRA2β binding, as would depleting SRSF10 or overexpressing hTRA2β.

1C8 has little impact on cell growth. Given that fairly large concentrations of 1C8 are required to affect *BCLAF1, GLYR1, CHEK1* and *SMN2* splicing, we sought to determine whether 1C8 had a more global effect on alternative splicing. First, we carried out endpoint RT-PCR on a set of 92 alternative splicing events. At 10 μM of 1C8, only one event (*INF2*) shifted with a *q*-value below 0.01 (Supplementary Table S1 and Figure S3). At 1 μM of 1C8, a concentration that severely blocks HIV replication, no alternative splicing event from this set was significantly altered (*q*-value ≤ 0.05).

We also carried out a transcriptome analysis by performing RNA-Seq on poly-dT selected RNA from the HeLa-HIV cells treated with increasing concentrations of 1C8 (0, 1, 5, 10 and 20 μM) in quadruplicates. The raw fastq files and MISO analysis results are available at GEO accession number GSE-76947. We used the MISO software (47) to investigate thousands of alternative splicing events. The MISO analysis revealed 86 alternatively spliced events affected by 30 or more percentage points with a Bayesian coefficient score >20 in at least one level of drug treatment (Supplementary Figure S4 and Supplementary Table S2). All replicates were pooled for each drug treatment level as the MISO pipeline does not account for replicates. We selected 22 of these units with appropriate amplicon sizes for validation by endpoint RT-PCR using 0, 1, 5 and 10 μM of 1C8 on HeLa-HIV cells. Of the 22 units, five were confirmed as hits (*DOM3Z, ESYT2, ITGB3BP, RBM41, SPTAN1*; Supplementary Figure S5). To determine if these splicing units are controlled by SRSF10, we knocked down SRSF10 in HeLa-HIV cells. Three units (*ITGB3BP, RBM41* and *SPTAN1*) reacted to the depletion of SRSF10 with a shift in splicing in the same direction as a treatment of HeLa-HIV cells with 1C8 (Supplementary Figure S6). Although 10 μM of 1C8 shifted the above six units between 13 and 45 percentage points, the 1 μM concentration of 1C8 produced changes between 0 and 12 percentage points (Supplementary Figure S4), representing values that are close to or below our validation threshold of 10 percentage points. Our results suggest that 1 μM of 1C8, which has a strong effect on HIV-1 replication, has a relatively minor impact on the alternative splicing of cellular pre-mRNAs.

We further examined our RNA-Seq data to determine the impact of 1C8 on the levels of cellular transcripts. At 20 μM of 1C8, approximately 1500 genes were significantly differentially expressed; half of which showed an increase in expression >2-fold (Supplementary Figure S7). At 1 μM of 1C8, <150 genes were affected (Supplementary Table S3), and only a handful exhibited a change in expression >4-fold. While the distribution of differentially expressed genes seems to favor those that are lowly expressed (Supplementary Figure S8), RT-PCR validation would be required to confirm that these are all true differences and not merely a reflection of the greater impact of small changes on less abundant transcripts.

## DISCUSSION

The discovery of new molecules displaying anti-HIV activity can inform us about HIV-1 biology and has the potential to improve our capacity to combat drug-resistant strains of HIV-1. Although targeting a molecular mechanism used by the host increases the risk of toxicity, the reliance of the virus for this mechanism may lead to HIV-1 hypersensitivity and offer a therapeutic window that circumvents host toxicity. Such an outcome may be possible when a compound alters the activity of a regulator, or a combination of regulators involved in the production of multiple viral components. The HIV-1 primary transcript is alternatively spliced to produce several distinct viral mRNAs that are essential for productive HIV-1 replication. This stringent requirement may explain why compounds that completely abrogate HIV-1 replication like IDC16 or digoxin, only have moderate toxicity in cell culture or in patients suffering from other diseases (14,16).

Our efforts to increase the arsenal of anti-HIV compounds led us to 1C8, which abrogates replication of various HIV strains, including those displaying resistance against current anti-HIV-1 drugs (17). 1C8 reduces transcription from the HIV promoter, and alters the splicing of viral introns. Since HIV-1 replication is strongly inhibited with 1 μM of 1C8, a concentration that has a significant, yet low impact on HIV-1 transcription and splicing, we propose that the accumulation of minor imbalances in viral expression severely compromises the production of virions.

The original indole compound IDC16 that displays anti-HIV activity was found to inactivate splicing enhancers controlled by SRSF1 (15). The diheteroarylamide-based compound 1C8 did not interfere with the splicing modulating activity of SRSF1, or the related SR protein SRSF9. Rather, 1C8 neutralized the ability of ectopically expressed SRSF10 to modulate 5΄ splice site selection on *Bcl-x* transcripts. The splicing of *Bcl-x* is controlled by at least 20 different RNA binding proteins (48), yet 1C8 does not affect *Bcl-x* splicing in normally growing cells. Only when cells are treated with oxaliplatin, and SRSF10 is co-opted into function (31), does 1C8 abrogate the oxaliplatin-mediated splicing shift of endogenous *Bcl-x*. High concentrations of 1C8 also shift the alternative splicing of cellular transcripts of *BCLAF1, ITGB3BP, RBM41, SPTAN1, SMN2, CHEK1* and *GLYR1* in the same direction as a siRNA-mediated depletion of SRSF10. Overall, these results support the view that 1C8 alters the function of SRSF10. For HIV-1, the depletion of SRSF10 mimicked the 1C8-induced Tat1/Nef2 change in splice site selection, and reduced the splicing efficiency of several viral introns. The only example where the outcome of 1C8 and of the depletion of SRSF10 differed was in the case of HIV-1 D3/A3 splicing, which was stimulated by 1C8. Since D3/A3 splicing is stimulated by hTRA2β (43), and that 1C8 strongly increases the interaction between SRSF10 and hTRA2β, we propose that 1C8 stimulates the assembly of an enhancer complex comprising these two factors. Consistent with this model, the depletion of SRSF10 abrogates the 1C8-mediated splicing stimulation of D3/A3.

SRSF10 lacks the second RRM homology domain present in SRSF1, and has a substantially larger RS domain that can be divided in the RS1 and RS2 portions (Figure 6). Phosphorylation is known to control the activity SRSF10 (40,42,49). Treating cells with 1C8 promotes the dephosphorylation of phosphoserine 133 in the RS1 domain of SRSF10. Serine 133 is important for the activity of SRSF10 (31). Although DNA damage by oxaliplatin dephosphorylates serine 133 (31), 1C8 does not elicit DNA damage, but rather antagonizes the oxaliplatin-induced splicing shift on endogenous *Bcl-x*. Although sharing an impact on S133, oxaliplatin and 1C8 likely promote additional and potentially distinct dephosphorylation events at other positions on SRSF10. At this stage, it is unclear if 1C8 stimulates the activity of phosphatases (e.g. PP1) or represses the activity of specific kinases (such as CLK1, SRPK1 and SRPK2) that act on SRSF10 (49). However, a general impact of 1C8 on SR protein kinases is unlikely given that the activities of SRSF1 and SRSF9 are not affected. Alternatively, 1C8 may inhibit the activity of SR kinases only after they interact with SRSF10. Blocking their activity at a kinetic intermediate stage may increase the time SRPK1 and SRPK2 spend in association with SRSF10, explaining why 1C8 increases the recovery of SRPK1 and SRPK2 in association with SRSF10. The relatively low number of cellular alternative splicing events affected by 1C8 is also consistent with specificity of action because a general alteration in the activity of kinases that target SR proteins are expected to have a broad impact on cellular pre-mRNA splicing (50).

Thus, our observations suggest that 1C8 alters the regulatory function of SRSF10 on HIV-1 splicing and that this impacts HIV-1 expression and replication. 1C8 decreases the splicing of some viral introns, while it stimulated D3/A3 splicing, possibly by altering interactions with other splicing regulators, such as hTRA2β. Notably, we also showed that decreased SRSF10 function affects the expression of Tat as well as of the late viral proteins Gag and Env that are produced from incompletely spliced HIV-1 mRNAs, consistent with the view that the accumulation of small splicing differences can ultimately impact viral replication.

In addition to altering viral splicing, 1C8 also affected the steady-state levels HIV-1 mRNAs suggesting an impact on transcription, that was not reproduced by the depletion of SRSF10. In addition, it is unclear if the cytoplasmic degradation or the defective transport of unspliced HIV-1 mRNAs elicited by 1C8 is linked to SRSF10 or to other factors whose activities may be altered by 1C8. Likewise, 1C8 affected the alternative splicing of two units that were insensitive to the depletion of SRSF10, suggesting that 1C8 may affect the activity of other splicing factors. Our analysis of the impact of 1C8 on the alternative splicing of cellular RNAs indicated that relatively high concentrations (10 and 20 μM) of 1C8 were required to produce robust splicing shifts (13-45 percentage points) on a relatively small number of events. At 1 μM of 1C8, splicing shifts were either not occurring or were less than 13 percentage points. Although we cannot rule out that some events in cellular transcripts not detected in our analysis may be more strongly affected by 1C8, the low cytoxicity of 1C8 suggests a minimal impact on cellular processes, including alternative splicing. Overall, our results therefore indicate that a low dose of 1C8 may provide sufficient cumulative impact to efficiently inhibit HIV-1 replication, while alterations of cellular events may remain limited.

## Supplementary Material

Supplementary Data
