# Supplementary material for: Modulation of the splicing regulatory function of SRSF10 by a novel compound that impairs HIV-1 replication
Source: Nucleic Acids Res. 2016 Dec 7;45(7):4051–67. doi: 10.1093/nar/gkw1223 (PMC5397194; doi:10.1093/nar/gkw1223)
Supplement: Supplementary Data [file gkw1223_Supp.pdf]

SUPPLEMENTARY INFORMATION

Supplementary Figure S1. Effect of 1C8 on viral RNA levels in the 24NLESG cell line.

Schematic of HIV-1 provirus in the 24NLESG cell line is shown on top. HIV-1 was induced in 24NELSG cells by addition of PMA in the presence of DMSO or 10 µM 1C8. After 24 h, cells were harvested and total RNA extracted. HIV-1 unspliced (US) RNA levels were assayed using the Aurum Total RNA Lysis Kit (Bio-Rad), as per manufacturer's instructions with the addition of Turbo DNase (Ambion). Purified RNA (0.5-2 μg) was reverse transcribed using M-MLV (Invitrogen). HIV-1 and actin mRNA levels in DMSO- and compound-treated samples were quantified by qPCR using the Mastercycler ep realplex (Eppendorf). Gene quantification was evaluated using the absolute quantification method, normalized to β-actin expression, and expressed relative to DMSO treatment.

Supplementary Figure S2. 1C8 does not affect hTRA2β and hnRNP F protein expression.

Western blot analysis of HeLa-HIV cells treated with different concentrations of 1C8. Protein extracts were fractionated by SDS-PAGE and transferred to a nitrocellulose membrane that was decorated with anti-hTRA2β and hnRNP F antibodies.

Supplementary Figure S3. RT-PCR analysis of splicing changes induced by 1C8.

Scatter plot comparing the percent splicing index (PSI) for 96 alternative splicing events in HeLa-HIV cells treated (*y*-axis) or not (*x*-axis) with 10 µM of 1C8. Values were obtained by quantitating amplification products obtained endpoint RT-PCR and fractionated on a Caliper LC-90 automated microfluidic station. Each point represents an average of triplicates with standard variations shown. Diagonals that run parallel to the central diagonal depict a ± 10% PSI window. *q*-values for the 1C8-induced difference are shown in Supplementary Table S1. The red dot represents the only splicing event (*INF2)* that is shifted with a *q*-value ≤ 0.01. White dots represent events with *q*-values ≤ 0.05 as listed in Supplementary Table S1.

Supplementary Figure S4. RNA-Seq analysis of splicing changes induced by 1C8.

Scatter plot comparing the percent splicing index (PSI) of more than 62 000 alternative splicing events in HeLa-HIV cells not treated (*x*-axis) or treated (*y*-axis) with 1, 5, 10 and 20 µM of 1C8. Diagonals that run parallel to the central diagonal depict a ± 30% PSI window. All replicates of a given treatment were merged into a single file before MISO analysis of RNA-Seq data. The analysis was performed for all concentrations of 1C8, revealing a total of 86 unique events significantly affected (red dots indicating ∆PSI larger than 30 percentage points and with Bayesian coefficient superior to 20 ; see Supplementary Table S2 for a list of significant events).

Supplementary Figure S5. Validation of five cellular splicing events affected by 1C8.

A. Graphic presentation of the splicing unit (bottom of the left panel) and the results of the RNA-Seq and MISO analysis for *ITGB3BP* unit from samples of HeLa-HIV cells treated with 1C8 (top of the left panel). The percent splicing index (PSI) of the *ITGB3BP* unit was determined by endpoint RT-PCR on samples of HeLa-HIV cells treated with the indicated concentrations of 1C8 and these values are shown in the histograms of the right panel. **B-E.** Similar descriptions for the alternative splicing units of *DOM3Z, ESYT2, RBM41* and *SPTAN1*, respectively.

Supplementary Figure S6. Impact of siRNA-mediated knockdown of SRSF10 on five cellular splicing events affected by 1C8.

A. Immunoblot showing the siRNA-mediated depletion of SRSF10 in 293 cells**.** (**B-F**) Histograms with percent splicing index (PSI) values for splicing units in *ITGB3BP, DOM3Z, ESYT2, RBM41* and *SPTAN1*.

Supplementary Figure S7. MA plots depicting expression changes (log2FC) of human genes caused by different concentrations of 1C8 relative to their relative expression levels (logCPM) in untreated samples.

The plots contain all expressed genes (15 867) based on a cpm cutoff of >1 in at least 4 experimental samples. All the MA plots are on the same scale (fold change from +4 to -4). Genes whose expression increases and decreases by more than 2-fold are in red and blue, respectively.

Supplementary Figure S8. Bar plots of expression hits at 1 μm of 1C8.

The distribution of genes whose expression changes. Approximately 4000 expressed genes were binned in each category according to their global expression level in untreated samples (low, medium, high and super high). The number of genes whose expression was affected (increased in red and repressed in blue) at least two-fold is represented by bar graphs.

Supplementary Table S1. Alternative splicing of cellular transcripts affected by 1C8 as measured by endpoint RT-PCR.

A set of 92 events was analyzed for alternative splicing using endpoint RT-PCR. Genes were selected for their ability to yield two amplicons of sizes that provide adequate resolution by microfluidic analysis using a RT-PCR screening platform (sequences of forward and reverse primers are indicated). HeLa-HIV cells were treated with 1C8 (1 and 10 μM) or DMSO as a control (in triplicates). Total RNA was extracted and RT-PCR analysis was carried out. For each sample, percent splicing index (PSI) values are given and changes relative to the control (ΔPSI) were calculated. We used a statistical approach tailored to the analysis of genomic data based on the false discovery rate (FDR) expressed as a *q*-value. For example, a 5% FDR, *q* value ≤ 0.05 indicates that 5% of the results judged to be significant could represent false positive signals. The section in green indicates *q* values that are smaller than 0.05, while only one value (in red) is inferior to 0.01.

Supplementary Table S2. Cellular transcripts whose alternative splicing are affected by 1C8 as revealed by MISO analysis of RNA-Seq data.

MISO analysis of RNA-Seq data revealed a set of 86 alternative splicing units affected by more than 30 percentage points in samples of HeLa-HIV cells treated with 1C8 (Bayesian coefficient score greater than 20) in at least one level of drug treatment. Essentially, 106 significant shifting events were discovered respectively distributing as 9, 16, 25 and 56 in the 1, 5, 10 and 20 μM treatment with 1C8 (20 shifting events are occurring at more than one concentration). Event types are alternative 3'splice site (A3SS) alternative 5' splice site (A5SS) and skipped exon (SE), retained intron (RI) and mutually exclusive exon (MXE).

Supplementary Table S3. Cellular transcripts with expression affected by 1 μM of 1C8 based on RNA-Seq data.

List of transcripts whose expression was affected more than 2-fold ($\log_2$ greater than 1 or smaller than -1) with FDR values inferior to 0.05.

Supplementary Table S4. Primers used in the study.

Gene	*q*-values of ΔPSI	
	CTLR – 1 μM 1C8	CTLR – 10 μM 1C8
INF2	6.470E-1	4.171E-3
CLK1	7.744E-1	1.283E-2
AP2B1	5.116E-1	1.719E-2
ABI1	5.116E-1	3.226E-2
ADARB1	6.102E-1	3.226E-2
EPB41L1	8.901E-1	3.228E-2
GRHL1	6.470E-1	3.228E-2
ADARB1	6.102E-1	3.298E-2
AMACR	6.102E-1	3.298E-2
ATG16L1	9.847E-1	3.298E-2
C3orf17	9.847E-1	3.298E-2
ECT2	9.991E-1	3.298E-2
KCNAB2	8.901E-1	3.298E-2
MAPKAP1	8.901E-1	3.298E-2
SMN2	6.102E-1	3.298E-2
HNRNPAB	8.040E-1	4.118E-2
ASB3	5.116E-1	4.425E-2
PALM	7.711E-1	4.425E-2
CASC4	6.102E-1	4.747E-2
ZMIZ2	9.991E-1	4.747E-2
APC	9.991E-1	4.763E-2
BRD8	9.991E-1	4.763E-2
DCUN1D4	8.040E-1	4.763E-2
NKTR	9.991E-1	4.763E-2
BRD8	9.314E-1	4.980E-2
LRP8	7.744E-1	4.980E-2
PARL	6.470E-1	5.107E-2
LLGL2	7.744E-1	5.219E-2
LONRF3	8.040E-1	5.267E-2
TBC1D25	7.711E-1	5.349E-2
PLOD2	8.794E-1	5.471E-2
GEMIN7	9.847E-1	5.650E-2
MKNK1	9.847E-1	6.202E-2
OSBPL3	8.040E-1	6.202E-2
NFAT5	6.470E-1	6.303E-2
PPIP5K1	7.744E-1	6.951E-2
AGAP1	9.991E-1	7.385E-2
MTMR3	8.040E-1	7.385E-2
BOLA3	6.040E-1	7.435E-2
MBD1	6.470E-1	7.488E-2
CLCN6	6.470E-1	9.172E-2
FBF1	8.794E-1	9.466E-2
POLB	9.702E-1	9.466E-2
PML	6.244E-1	9.800E-2
CAMKK2	7.744E-1	9.829E-2
ODF2L	9.991E-1	9.829E-2

Gene	*q*-values of ΔPSI	
	CTLR – 1 μM 1C8	CTLR – 10 μM 1C8
ARFIP1	7.076E-1	1.028E-1
ERBB2IP	9.991E-1	1.028E-1
GTF2H2C	8.794E-1	1.028E-1
POGZ	9.847E-1	1.028E-1
ATG5	5.116E-1	1.046E-1
CDK5RAP2	6.102E-1	1.072E-1
NDEL1	7.744E-1	1.072E-1
NFAT5	5.116E-1	1.072E-1
DPP8	7.744E-1	1.131E-1
GAB1	9.314E-1	1.201E-1
ARHGEF11	7.076E-1	1.416E-1
FAM86A	7.711E-1	1.494E-1
AKIP1	7.556E-1	1.586E-1
GIT2	9.991E-1	1.586E-1
INSR	6.102E-1	1.586E-1
TMEM218	9.991E-1	1.586E-1
EIF6	5.116E-1	1.834E-1
PAOX		1.843E-1
MRPL33	8.040E-1	1.922E-1
PITPNC1	5.116E-1	1.922E-1
ATP2A1	7.744E-1	1.993E-1
GTF2I	9.012E-1	2.018E-1
SYNE2	6.040E-1	2.018E-1
AP3M1	7.711E-1	2.226E-1
N6AMT1	9.991E-1	2.226E-1
BTRC	8.673E-1	2.231E-1
ATP5S	7.744E-1	2.334E-1
ADNP	9.991E-1	2.361E-1
CD151	5.116E-1	2.467E-1
CTBP1	8.927E-1	2.467E-1
LRRC23	8.040E-1	2.467E-1
ABCD4	7.711E-1	2.617E-1
MEIS2	5.116E-1	2.668E-1
LHX6	6.470E-1	2.874E-1
HPS1	9.770E-1	3.180E-1
OSBPL9	7.744E-1	3.312E-1
MCAT	9.277E-1	3.323E-1
DTNB	9.991E-1	3.662E-1
MTMR2	9.314E-1	3.662E-1
NFATC2		3.710E-1
ANAPC11	5.116E-1	4.092E-1
MARK2	9.036E-1	4.101E-1
ARHGAP17	8.040E-1	4.104E-1
MAPKAP1	9.991E-1	4.104E-1
PDE9A	8.040E-1	4.145E-1
BTC	5.116E-1	4.310E-1

Supplementary TABLE S1

Gene Symbol	Event type	Treatment	Psi. Diff.Score	Bayes Factor	Event name
AC005077.12	A3SS	10uM	0.40	660327.00	chr7:76090972:76091153:+@chr7:76092862:76093026:+@chr7:76109738:76110094:+@chr7:76111825:76112464:+
AC068533.7	A5SS	5uM	0.30	638.00	chr7:65592691:65592727:+@chr7:65595114:65595235:+@chr7:65595731:65595829:+
ALG13	A5SS	1uM	0.34	67080.00	chrX:110928193:110928331:+@chrX:110932315:110932425:+@chrX:110951255:110951621:+@chrX:110952193:110952276:
ARFGAP2	MXE	10uM	-0.31	21.00	chr11:47198067:47198185:-@chr11:47197402:47197474:-@chr11:47196733:47196864:-@chr11:47193832:47193884:-
ARFGAP2	MXE	20uM	-0.30	425.00	chr11:47198067:47198185:-@chr11:47197402:47197474:-@chr11:47196733:47196864:-@chr11:47193832:47193884:-
ARL13B	A3SS	10uM	0.36	20.00	chr3:93698983:93699326:+@chr3:93714722:93714788:+@chr3:93754175:93754285:+@chr3:93755396:93755598:+
ASAH2B	A3SS	5uM	-0.32	29.00	chr10:52502675:52502770:+@chr10:52504962:52505034:+@chr10:52509103:52509162:+
ATP5G2	SE	20uM	-0.35	54.00	chr12:54069960:54070512:-@chr12:54067480:54067596:-@chr12:54066360:54066429:-
ATPAF1	A5SS	1uM	0.36	158.00	chr1:46655029:46654852|46654914:-@chr1:46654353:46654652:-
ATPAF1	A5SS	20uM	0.30	29.00	chr1:46655029:46654852|46654914:-@chr1:46654353:46654652:-
BTD	MXE	5uM	-0.39	39.00	chr3:15643255:15643401:+@chr3:15643644:15643817:+@chr3:15676931:15677195:+
C19orf47	SE	20uM	0.41	248.00	chr19:40839758:40839836:-@chr19:40838429:40838550:-@chr19:40834320:40834457:-
C19orf47	SE	5uM	0.42	299.00	chr19:40839758:40839836:-@chr19:40838429:40838550:-@chr19:40834320:40834457:-
CAMK2D	A5SS	10uM	-0.51	1.00E+12	chr4:114378719:114378447|114378491:-@chr4:114372188:114375671:-
CAMK2D	A5SS	20uM	-0.43	8.75E+06	chr4:114378719:114378447|114378491:-@chr4:114372188:114375671:-
CAMK2D	A5SS	5uM	-0.44	406.00	chr4:114378719:114378447|114378491:-@chr4:114372188:114375671:-
CC2D1B	RI	1uM	-0.31	12805.00	chr1:52824133-52824003:-@chr1:52823570-52823453:-
CNOT1	MXE	20uM	0.31	49.00	chr16:58663632:58663790:-@chr16:58658577:58658698:-@chr16:58657186:58657313:-@chr16:58633140:58633415:-
DCHS1	A3SS	20uM	0.32	31.00	chr11:6624634:6624880:-@chr11:6623445|6623494:6623082:-
DDX41	RI	20uM	0.33	1.00E+12	chr5:176942822-176942686:-@chr5:176942340-176942187:-
DHX34	SE	1uM	0.39	81.00	chr19:47879700:47879817:+@chr19:47880172:47880246:+@chr19:47880357:47880463:+
DOM3Z	A3SS	20uM	0.40	76764.00	chr6:31938689:31938924:-@chr6:31938529|31938602:31938383:-
EDC3	SE	20uM	0.34	530.00	chr15:74988221:74988386:-@chr15:74979432:74979520:-@chr15:74967302:74967483:-
ELP5	SE	10uM	-0.35	1.00E+12	chr17:7157902:7158122:+@chr17:7160176:7160357:+@chr17:7161907:7162002:+
ENGASE	A5SS	20uM	-0.31	1.00E+12	chr17:77077980:77078145|77078959:+@chr17:77079102:77079205:+
ERBB2	SE	10uM	0.33	32.00	chr17:37868575:37868701:+@chr17:37869406:37869522:+@chr17:37871539:37871612:+
ESYT2	SE	10uM	-0.31	1.00E+12	chr7:158552177:158552272:-@chr7:158545472:158545534:-@chr7:158542340:158542414:-
ESYT2	SE	20uM	-0.31	1.00E+12	chr7:158552177:158552272:-@chr7:158545472:158545534:-@chr7:158542340:158542414:-
FGFR1	SE	20uM	0.38	91.00	chr8:38314874:38315052:-@chr8:38287200:38287466:-@chr8:38285864:38285953:-
FN1	SE	20uM	0.36	1.00E+12	chr2:216259251:216259442:-@chr2:216257654:216257926:-@chr2:216256355:216256537:-
GALT	A5SS	10uM	0.41	1.00E+12	chr9:34646674-34646783:+@chr9:34647086-34647255:+
GALT	A5SS	20uM	0.33	2003.00	chr9:34646642:34646783|34646916:+@chr9:34647086:34647255:+
GALT	A5SS	20uM	0.35	2.28E+06	chr9:34646674-34646783:+@chr9:34647086-34647255:+
GALT	A5SS	5uM	0.36	2.32E+07	chr9:34646642:34646783|34646916:+@chr9:34647086:34647255:+
GALT	A5SS	5uM	0.31	2216.00	chr9:34646674-34646783:+@chr9:34647086-34647255:+
GUSB	SE	5uM	0.34	41.00	chr7:65445211:65445396:-@chr7:65444821:65444898:-@chr7:65444386:65444528:-
HDAC6	RI	10uM	0.31	778342.00	chrX:48664038-48664078:+@chrX:48664775-48664871:+
HDLBP	SE	20uM	-0.31	76.00	chr2:242254985:242255117:-@chr2:242208244:242208710:-@chr2:242207892:242207956:-
IL17RC	RI	20uM	0.45	340.00	chr3:9965565-9965704:+@chr3:9965916-9965975:+
IRF9,RNF31	SE	10uM	0.35	24.00	chr14:24633823:24634164:+@chr14:24634266:24634392:+@chr14:24635056:24635171:+
IRF9,RNF31	SE	20uM	0.31	20.00	chr14:24633823:24634164:+@chr14:24634266:24634392:+@chr14:24635056:24635171:+
ITGB3BP	SE	10uM	-0.34	1.00E+12	chr1:63919589:63919645:-@chr1:63913236:63913285:-@chr1:63912477:63912527:-
ITGB3BP	SE	20uM	-0.42	1.00E+12	chr1:63919589:63919645:-@chr1:63913236:63913285:-@chr1:63912477:63912527:-
KIAA0528	SE	20uM	0.32	52.00	chr12:22612426:22612476:-@chr12:22611418:22611519:-@chr12:22609905:22610095:-
KIF21A	SE	20uM	-0.31	101.00	chr12:39713708:39713815:-@chr12:39711875:39712003:-@chr12:39705219:39705355:-
LAS1L	SE	10uM	0.35	1.00E+12	chrX:64744845:64744930:-@chrX:64744444:64744494:-@chrX:64743936:64744142:-
LAS1L	SE	20uM	0.35	1.00E+12	chrX:64744845:64744930:-@chrX:64744444:64744494:-@chrX:64743936:64744142:-
LINC00665	MXE	20uM	0.31	68.00	chr19:36822349:36822620:-@chr19:36821155:36821394:-@chr19:36813032:36813286:-@chr19:36812895:36812932:-
LMBR1L	MXE	1uM	0.44	93973.00	chr12:49500744:49500828:-@chr12:49499707:49499740:-@chr12:49498529:49498668:-@chr12:49497470:49497596:-
LTBP1	SE	20uM	0.31	72.00	chr2:33540211:33540336:+@chr2:33567905:33568030:+@chr2:33572434:33572577:+
MEGF6	SE	5uM	0.40	3020.00	chr1:3413797:3413925:-@chr1:3413552:3413683:-@chr1:3413219:3413347:-
MLL3	A5SS	1uM	-0.36	30.00	chr7:151842380:151842238|151842250:-@chr7:151841798:151841966:-
MTMR14	SE	1uM	-0.32	84.00	chr3:9730628:9730766:+@chr3:9739395:9739550:+@chr3:9743474:9744078:+
MTMR2	SE	10uM	0.41	142.00	chr11:95657039:95657371:-@chr11:95647406:95647476:-@chr11:95621320:95621425:-
MTMR2	SE	20uM	0.33	36.00	chr11:95657039:95657371:-@chr11:95647406:95647476:-@chr11:95621320:95621425:-
MTMR2	SE	20uM	0.31	131.00	chr11:95621320:95621425:-@chr11:95620776:95620848:-@chr11:95598765:95598840:-
MUC1	MXE	5uM	-0.32	50.00	chr1:155160484:155160539:-@chr1:155160198:155160329:-@chr1:155159931:155160052:-@chr1:155159701:155159850:-
NAA60	MXE	10uM	-0.37	138.00	chr16:3493668:3493837:+@chr16:3497482:3497620:+@chr16:3498471:3498540:+
NAA60	MXE	10uM	-0.36	1.09E+10	chr16:3493649:3493827:+@chr16:3497482:3497620:+@chr16:3498471:3498540:+
NAA60	MXE	20uM	-0.34	16042.00	chr16:3493668:3493837:+@chr16:3497482:3497620:+@chr16:3498471:3498540:+
NAA60	MXE	5uM	-0.37	1205.00	chr16:3493668:3493837:+@chr16:3497482:3497620:+@chr16:3498471:3498540:+
NAA60	MXE	5uM	-0.37	1.00E+12	chr16:3493649:3493827:+@chr16:3497482:3497620:+@chr16:3498471:3498540:+
NAP1L4	SE	20uM	-0.35	1.00E+12	chr11:2972489:2972545:-@chr11:2970457:2970494:-@chr11:2965660:2966876:-
NDUFV3	MXE	10uM	-0.30	1.00E+12	chr21:44317037:44317157:+@chr21:44323292:44324386:+@chr21:44328974:44329773:+
NIPA2	MXE	20uM	0.36	992.00	chr15:23034147:23034427:-@chr15:23033894:23034006:-@chr15:23027801:23027922:-@chr15:23021198:23021429:-
NIPA2	MXE	20uM	0.37	2.20E+08	chr15:23034147:23034427:-@chr15:23033894:23034006:-@chr15:23033278:23033413:-@chr15:23021198:23021429:-
NIPA2	MXE	20uM	-0.41	27.00	chr15:23034147:23034427:-@chr15:23033894:23034006:-@chr15:23033278:23033413:-

Supplementary TABLE S2

<table>
<tr><th>Gene Symbol</th><th>Event type</th><th>Treatment</th><th>Psi. Diff.Score</th><th>Bayes Factor</th><th>Event name</th></tr>
<tr><td>PCM1</td><td>SE</td><td>10uM</td><td>0.36</td><td>172.00</td><td>chr8:17797200:17797370:+@chr8:17797551:17797667:+@chr8:17804695:17804872:+</td></tr>
<tr><td>PDCD10</td><td>MXE</td><td>10uM</td><td>-0.41</td><td>6.81E+06</td><td>chr3:167452594:167452651:-@chr3:167452002:167452034:-@chr3:167443189:167443261:-@chr3:167437850:167438061</td></tr>
<tr><td>PDCD10</td><td>MXE</td><td>10uM</td><td>-0.36</td><td>41052.00</td><td>chr3:167452002:167452138:-@chr3:167443189:167443261:-@chr3:167437850:167438061:-</td></tr>
<tr><td>PDCD10</td><td>MXE</td><td>20uM</td><td>-0.36</td><td>243.00</td><td>chr3:167452594:167452651:-@chr3:167452002:167452034:-@chr3:167443189:167443261:-@chr3:167437850:167438061</td></tr>
<tr><td>PLSCR3</td><td>SE</td><td>20uM</td><td>0.35</td><td>249.00</td><td>chr17:7296786:7296828:-@chr17:7296463:7296683:-@chr17:7296110:7296271:-</td></tr>
<tr><td>PPIP5K2</td><td>SE</td><td>20uM</td><td>-0.30</td><td>13821.00</td><td>chr5:102515722:102515889:+@chr5:102518935:102519108:+@chr5:102520373:102520445:+</td></tr>
<tr><td>PREPL</td><td>SE</td><td>20uM</td><td>0.36</td><td>1372.00</td><td>chr2:44588663:44589001:-@chr2:44586636:44586889:-@chr2:44573407:44573529:-</td></tr>
<tr><td>RBM23</td><td>SE</td><td>10uM</td><td>0.34</td><td>19811.00</td><td>chr14:23388208:23388358:-@chr14:23380995:23381112:-@chr14:23380537:23380612:-</td></tr>
<tr><td>RBM41</td><td>MXE</td><td>20uM</td><td>0.31</td><td>1.00E+12</td><td>chrX:106358582:106358786:-@chrX:106356627:106356698:-@chrX:106331666:106332069:-</td></tr>
<tr><td>RCOR3</td><td>SE</td><td>20uM</td><td>0.32</td><td>249.00</td><td>chr1:211477425:211477482:+@chr1:211486062:211486177:+@chr1:211486766:211489725:+</td></tr>
<tr><td>RHBDD2</td><td>SE</td><td>1uM</td><td>-0.35</td><td>37.00</td><td>chr7:75508317:75508578:+@chr7:75510683:75510804:+@chr7:75511147:75511554:+</td></tr>
<tr><td>RHBDD2</td><td>SE</td><td>20uM</td><td>-0.30</td><td>64.00</td><td>chr7:75508317:75508578:+@chr7:75510683:75510804:+@chr7:75511147:75511554:+</td></tr>
<tr><td>RP4-564F22.2</td><td>A5SS</td><td>20uM</td><td>0.32</td><td>1.00E+12</td><td>chr20:37063976:37063670|37063840:-@chr20:37063147:37063269:-</td></tr>
<tr><td>SCNN1D</td><td>SE</td><td>20uM</td><td>-0.51</td><td>35428.00</td><td>chr1:1219358:1219470:+@chr1:1220951:1221044:+@chr1:1221306:1221658:+</td></tr>
<tr><td>SCNN1D</td><td>SE</td><td>5uM</td><td>-0.38</td><td>40.00</td><td>chr1:1219358:1219470:+@chr1:1220951:1221044:+@chr1:1221306:1221658:+</td></tr>
<tr><td>SDR39U1</td><td>A5SS</td><td>20uM</td><td>-0.30</td><td>76.00</td><td>chr14:24911466:24911304|24911384:-@chr14:24910880:24911001:-</td></tr>
<tr><td>SDR39U1</td><td>A5SS</td><td>20uM</td><td>-0.30</td><td>115.00</td><td>chr14:24911466:24911315|24911384:-@chr14:24910880:24911001:-</td></tr>
<tr><td>SDR39U1</td><td>A5SS</td><td>20uM</td><td>-0.40</td><td>4.54E+09</td><td>chr14:24911466:24911315|24911384:-@chr14:24909989:24910132:-</td></tr>
<tr><td>SDR39U1</td><td>A5SS</td><td>20uM</td><td>-0.36</td><td>1.50E+10</td><td>chr14:24911466:24911304|24911384:-@chr14:24909989:24910132:-</td></tr>
<tr><td>SEC22C</td><td>MXE</td><td>10uM</td><td>0.37</td><td>27.00</td><td>chr3:42610357:42610565:-@chr3:42608236:42608328:-@chr3:42605257:42605472:-@chr3:42605015:42605178:-</td></tr>
<tr><td>SEPT7P2</td><td>SE</td><td>5uM</td><td>0.31</td><td>27.00</td><td>chr7:45786814:45786910:-@chr7:45786426:45786547:-@chr7:45784596:45784647:-</td></tr>
<tr><td>SGSH</td><td>MXE</td><td>1uM</td><td>-0.33</td><td>22.00</td><td>chr17:78190831:78190991:-@chr17:78188832:78188937:-@chr17:78188414:78188564:-@chr17:78187971:78188127:-</td></tr>
<tr><td>SGSM2</td><td>SE</td><td>10uM</td><td>-0.38</td><td>1440.00</td><td>chr17:2268509:2268635:+@chr17:2270565:2270699:+@chr17:2274556:2274709:+</td></tr>
<tr><td>SPTAN1</td><td>SE</td><td>10uM</td><td>-0.40</td><td>6076.00</td><td>chr9:131353756:131353904:+@chr9:131355262:131355321:+@chr9:131356454:131356652:+</td></tr>
<tr><td>ST3GAL6</td><td>MXE</td><td>5uM</td><td>-0.30</td><td>28.00</td><td>chr3:98491657:98491760:+@chr3:98492764:98492827:+@chr3:98503789:98503884:+@chr3:98506880:98507066:+</td></tr>
<tr><td>TAPBP</td><td>MXE</td><td>20uM</td><td>-0.31</td><td>3.73E+10</td><td>chr6:33281782:33282164:-@chr6:33281471:33281641:-@chr6:33280994:33281254:-@chr6:33272766:33273164:-</td></tr>
<tr><td>TOR1AIP1</td><td>A3SS</td><td>20uM</td><td>0.34</td><td>87.00</td><td>chr1:179853827:179853904:+@chr1:179858445|179858448:179858504:+</td></tr>
<tr><td>TPM1</td><td>A3SS</td><td>10uM</td><td>0.31</td><td>1444.00</td><td>chr15:63334838:63335142:+@chr15:63335668|63335905:63336030:+</td></tr>
<tr><td>TPM1</td><td>A3SS</td><td>20uM</td><td>0.43</td><td>1.00E+12</td><td>chr15:63334838:63335142:+@chr15:63335668|63335905:63336030:+</td></tr>
<tr><td>TPM1</td><td>A3SS</td><td>5uM</td><td>0.34</td><td>269.00</td><td>chr15:63334838:63335142:+@chr15:63335668|63335905:63336030:+</td></tr>
<tr><td>TRIM5</td><td>SE</td><td>20uM</td><td>0.34</td><td>115.00</td><td>chr11:5706098:5706339:-@chr11:5705216:5705369:-@chr11:5700991:5701468:-</td></tr>
<tr><td>UEVLD</td><td>MXE</td><td>10uM</td><td>0.36</td><td>27.00</td><td>chr11:18610160:18610293:-@chr11:18600271:18600355:-@chr11:18596894:18596959:-@chr11:18591761:18591924:-</td></tr>
<tr><td>WASF2</td><td>SE</td><td>20uM</td><td>-0.49</td><td>1.00E+12</td><td>chr1:27739066:27739221:-@chr1:27736186:27736700:-@chr1:27730734:27734840:-</td></tr>
<tr><td>WDR45</td><td>A3SS</td><td>20uM</td><td>-0.33</td><td>125.00</td><td>chrX:48935302:48935406:-@chrX:48934409|48934484:48934304:-</td></tr>
<tr><td>ZFYVE26</td><td>RI</td><td>20uM</td><td>0.35</td><td>1.17E+09</td><td>chr14:68229129-68228920:-@chr14:68228301-68228083:-</td></tr>
<tr><td>ZNF195</td><td>MXE</td><td>20uM</td><td>0.33</td><td>375.00</td><td>chr11:3392205:3392300:-@chr11:3383027:3383119:-@chr11:3381950:3382018:-@chr11:3379157:3381795:-</td></tr>
<tr><td>ZNF266</td><td>SE</td><td>20uM</td><td>0.37</td><td>28.00</td><td>chr19:9545784:9545869:-@chr19:9545474:9545535:-@chr19:9530741:9530894:-</td></tr>
<tr><td>ZNF330</td><td>A5SS</td><td>20uM</td><td>0.31</td><td>55.00</td><td>chr4:142143520:142143631|142143645:+@chr4:142145641:142145660:+</td></tr>
<tr><td>ZNF562</td><td>MXE</td><td>20uM</td><td>0.36</td><td>46.00</td><td>chr19:9771396:9771550:-@chr19:9770055:9770143:-@chr19:9768685:9768811:-@chr19:9767223:9767329:-</td></tr>
</table>

Supplementary TABLE S2

Gene	1 μM 1C8	
	Fold change (Log_2)	FDR value
GPR183	2.650443	2.48E-08
SPINK1	2.574378	7.71E-14
AC068057.1	2.149233	0.001587
INPP5D	2.075333	8.42E-09
KLHDC1	1.983792	0.003443
LPL	1.969363	4.94E-60
APOBEC3H	1.905028	1.13E-05
RP11-611O2.5	1.891939	0.000476
CHL1	1.840547	0.000369
EDN1	1.808004	5.82E-22
KCNN2	1.764675	2.13E-08
HSF2BP	1.724695	1.50E-47
FGB	1.674479	3.50E-06
RP11-809N8.5	1.658597	0.016428
TM4SF1	1.627575	2.11E-49
LIX1	1.626405	0.003474
HSPA9P1	1.603818	0.032998
RP5-1198O20.4	1.591723	2.08E-05
PDE2A	1.586868	4.06E-56
INSL4	1.579286	1.25E-21
ANO2	1.573054	1.57E-20
SCG2	1.557212	1.53E-06
PTGER3	1.527466	8.70E-08
RP11-807H7.2	1.483031	0.03631
CUL4A	1.468718	1.01E-43
C5orf46	1.415557	6.41E-09
CRISPLD2	1.406103	2.21E-11
RP11-115D19.1	1.403545	0.034854
GRHL3	1.400786	0.018813
TNFAIP3	1.398859	2.60E-39
RP11-78A19.4	1.394101	1.87E-05
ITGA11	1.377958	0.004201
FGG	1.37735	3.92E-09
FGFBP1	1.377072	0.00014
PSG5	1.342883	3.54E-05
HRCT1	1.341015	3.32E-11
CCDC68	1.303014	6.09E-20
SCG5	1.294259	5.36E-05
FTH1P11	1.292303	0.010688
MYT1L	1.290632	2.88E-11
AC023137.2	1.282045	0.001057
SPINK6	1.262633	0.0005
DNMT3L	1.247603	2.46E-08
PLEKHG4B	1.246211	0.018468
ALPK2	1.238146	2.20E-05
RGS2	1.231964	1.03E-23
SLC16A6	1.231223	1.23E-29
RP11-421L10.1	1.226661	0.008277
GLIPR1L2	1.218035	0.024921
RP11-144N1.1	1.21039	0.005083
RXFP1	1.208839	2.40E-05
VWA5A	1.207525	4.28E-33
ACTA2	1.202727	1.24E-05
RP1-140K8.5	1.188101	7.77E-09
RP11-399H11.2	1.185094	0.00139
PSG9	1.174724	0.009592
RP11-347C18.3	1.171276	0.040768
OLFM1	1.154358	3.80E-19
PSG1	1.151686	0.009213
CTC-295J13.3	1.150792	0.005046
CRB1	1.14419	1.19E-13
GAGE2A	1.142999	0.043287
FOXQ1	1.135102	1.02E-18
CYR61	1.12529	3.35E-24
EID3	1.124967	0.010763

Gene	1 μM 1C8	
	Fold change (Log_2)	FDR value
RP11-64D22.2	1.115672	5.69E-06
KB-1991G8.1	1.11473	0.001487
RP11-154J22.1	1.110004	0.00724
CD83	1.101461	2.12E-32
FMO5	1.100955	0.032603
SGK1	1.09107	4.27E-37
SPATA18	1.089338	1.11E-07
TM4SF1-AS1	1.086954	3.94E-06
CHKB-AS1	1.083088	0.019742
RP11-134G8.8	1.06926	0.040298
GRAMD3	1.068721	8.32E-11
MYPN	1.067613	3.75E-19
PCID2	1.062882	3.26E-32
CTD-2139B15.5	1.062001	3.21E-05
NUPR1	1.061691	5.55E-15
RP5-1028K7.2	1.060282	0.019401
FAM43A	1.059855	8.74E-15
CPM	1.05938	9.93E-22
CHMP1B	1.050854	5.77E-18
ARL4A	1.047929	2.43E-18
RP11-467D6.1	1.035512	0.000982
MFF	1.034311	1.47E-29
AC003104.1	1.030898	4.19E-06
SPINK13	1.028151	1.55E-07
ITPR1	1.025354	1.04E-17
RP11-356I2.4	1.015603	1.19E-08
LINC00471	1.008444	0.012322
SMC1B	1.003701	0.034351
FTH1P20	1.003479	0.026094
RP11-383H13.1	1.002233	0.040768
CFH	-1.00233	1.09E-05
RN7SK	-1.0043	0.040578
GPR50	-1.02022	2.83E-09
RP11-95O2.5	-1.02056	0.03365
EPGN	-1.02859	0.022321
AF003625.3	-1.03325	1.92E-05
KLRC2	-1.04006	4.89E-05
RP3-395M20.12	-1.04413	3.59E-05
HOXA-AS2	-1.05449	2.55E-15
RP11-715J22.2	-1.07652	0.025005
ITGB4	-1.09704	0.048179
PPL	-1.10616	2.23E-05
RP13-238F13.5	-1.12623	0.000755
ARC	-1.13624	0.003111
RAB6C-AS1	-1.14205	0.02
C20orf166-AS1	-1.14246	0.006623
LIPH	-1.15069	0.016412
RAPGEF3	-1.15633	6.11E-06
G0S2	-1.16014	0.003552
FAP	-1.17566	1.26E-15
GAS6-AS1	-1.18169	0.018203
ZCCHC12	-1.18399	2.50E-16
GRB7	-1.241	0.013022
ELF3	-1.25264	4.71E-10
RP11-284F21.9	-1.26778	6.16E-05
FOSB	-1.27149	7.83E-06
FAM231D	-1.27846	0.009667
H19	-1.28402	1.98E-05
CXCL3	-1.29749	0.001547
NMUR1	-1.38076	0.024921
SSC5D	-1.47675	1.95E-07
ANGPTL4	-1.62486	0.000903
RP11-474O21.5	-1.64184	0.000216
AL161626.1	-1.64267	0.034338
MIR663A	-1.72027	0.007235
LINC00319	-2.71392	2.49E-71

Supplementary TABLE S3

1. Primers for HIV-1

For analysis of HIV-1 transcripts, primers were designed based on Human immunodeficiency virus 1, complete genome sequence: NCBI Reference Sequence: NC_001802.1

1.1. Primers for quantitative real-time PCR

HIV-US.1.FWD	5'- CTG AAG CGC GCA CGG CAA -3'
HIV-US.1.REV	5'- GAC GCT CTC GCA CCC ATC TC -3'
HIV-US.2.FWD	5'- GGA CAG CTA CAA CCA TCC CT -3'
HIV-US.2.REV	5'- TGA CCT GAT TGC TGT GTC CT -3'
HIV-US.4.FWD	5'- GGG AAA GCT AGG GGA TGG TT -3'
HIV-US.4.REV	5'- TGA CCC AAA TGC CAG TCT CT -3'
HIV-SS.1.FWD	5'- GGC GGC GAC TGG AAG AAG C -3'
HIV-SS.1.REV	5'- CTA TGA TTA CTA TGG ACC ACA C -3'
HIV-SS.1.2.FWD	5'- GGC GGC GAC TGG AAG AAG C -3'
HIV-SS.1.2.REV	5'- CCC CCA TCT CCA CAA GTG CTG A -3'
HIV-SS.2.FWD	5'- GGC GGC GAC TGA CTC TGC TAT AAG -3'
HIV-SS.2.REV	5'- TTG GTC TTC TGG GGC TTG TT -3'
HIV-SS.3.FWD	5'- GGC GGC GAC TG T TTG TTT CAT AAC -3'
HIV-SS.3.REV	5'- AGA GAA GCT TGA TGA GTC TGA CTG -3'
HIV-SS.4.FWD	5'- GCG GCG ACT G AA TTG GGT GTC -3'
HIV-SS.4.REV	5'- GGC TGA CTT CCT GGA TGC TTC C -3'

1.2. Primers for HIV-1 endpoint PCR

HIV-AS-2.FWD	5'- TCT CTC GAC GCA GGA CTC G -3'
HIV-AS-2.REV	5'- TTG GGA GGT GGG TTG CTT TGA TAG -3'

2. Primers for *SRSF10* quantitative RT-PCR

FUSP1_G_1_F 5' AGAAAGTGGATTTGTGGACGGC 3'
FUSP1_G_1_R 5'-ACATTCCTCCCTTCCTTGGCTT-3'

3. Primers for quantitative RT-PCR of HIV fragments antibodies following RNA immunoprecipitation with anti-Flag

HIV_1-FWD	5'- GTGCAGAACATCCAGGGGCAAA-3'
HIV_1-REV	5'- TCTTGTGGGGTGGCTCCTTCT-3'
HIV_3-FWD	5'- TGTGGAAAGGAAGGACACCAAATGA-3'
HIV_3-REV	5'- TCCTTGTCTATCGGCTCCTGCT -3'
HIV_5-FWD	5'- CCCCGGCTGGTTTTGCGATT-3'
HIV_5-REV	5'- ACCTCTTCTTCTGCTAGACTGCCAT-3'
HIV_8-FWD	5'- CCCGACAGGCCCGAAGGAATA-3'
HIV_8-REV	5'- CCCCCTGCGTCCCAGAAGTT-3'

Supplementary TABLE S4

4. Primers for endpoint PCR of transcripts from *Bcl-x* minigenes

X34	5'-AGGGAGGCAGGCGACGGCGACGAGTTT-3'
X-Age-Rev	5'-CCGGTGGATCCCCCGGGCTGCAGGAATTCGAT-3'

5. Primers for endpoint PCR of various splicing units

BCLAF1-FWD	5'-GTCTGGTTCTGTTGGAAATA-3'
BCLAF1-REV	5'-AGCAAGCAGCCTGTCTTTAGTC-3'
GLYR1-FWD	5'-GCCTGGATCAAAGTGGAACA-3'
GLYR1-REV	5'-CTAGACTCCGGGATGGTGAG-3'
CHEK1-FWD	5'-GACTGGGACTTGGTGCAAAC-3'
CHEK1-REV	5'-TGCCATGAGTTGATGGAAGA-3'
SMN2-FWD	5'-CCTCCCATATGTCCAGATTCTC-3'
SMN2-REV	5'- GTGGTGTCATTTAGTGCTGCTC-3'
DOM3Z-FWD	5'-ATAGTGACGTGGCGGGGGCA-3'
DOM3Z-REV	5'-GAGAAGAGCAGAGGGTGGCTTC-3'
ESYT2-FWD	5'-TCAAAGCTGACAAAGACCAAGC-3'
ESYT2-REV	5'-TGGACAACAGGATTTGGGTTGC-3'
ITGB3BP-FWD	5'-AACAAAAACTGTTTGAAAAGAGTACAGG-3'
ITGB3BP-REV	5'-AAAATGGCTTTAAGGAATTCATAGCTGT-3'
NAP1L4-FWD	5'-CGAGGAGGGAGAAGACGAGGAT-3'
NAP1L4-REV	5'-GAAGTCCAGAGCTACAGGCAC-3'
RBM41-FWD	5'-TTTCAGGGAGCTGATCGTCACT-3'
RBM41-REV	5'-AGATCTCTTTCAGTCACCCGAGG-3'
SPTAN1-FWD	5'-GGTGGAAAGTGGAAGTGAACGA-3'
SPTAN1-REV	5'-AACCTGCTCCAAGTCTGCTCC-3'

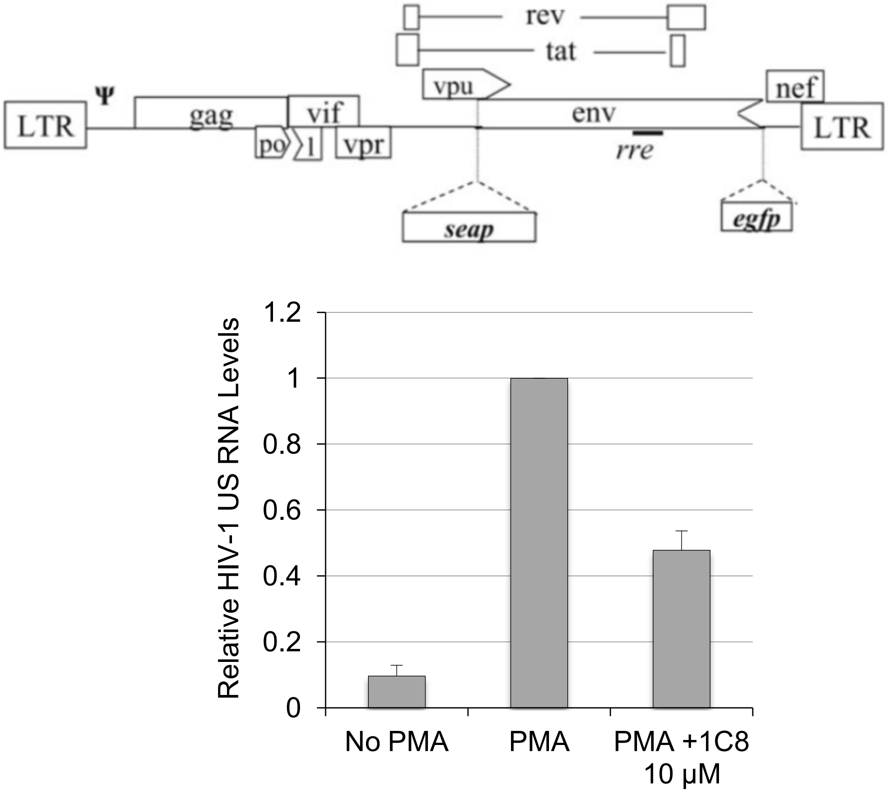

Supplementary Figure S1

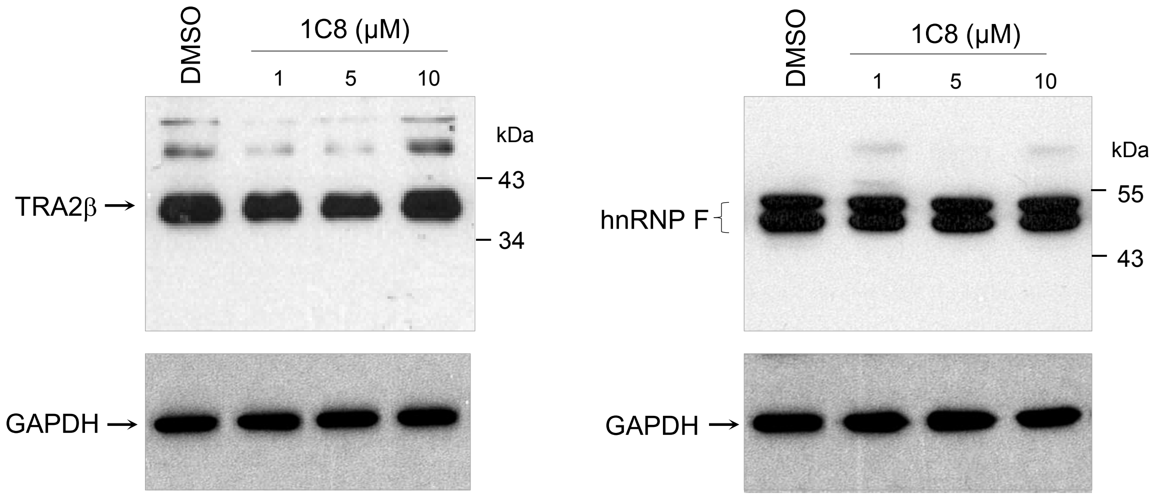

Supplementary Figure S2

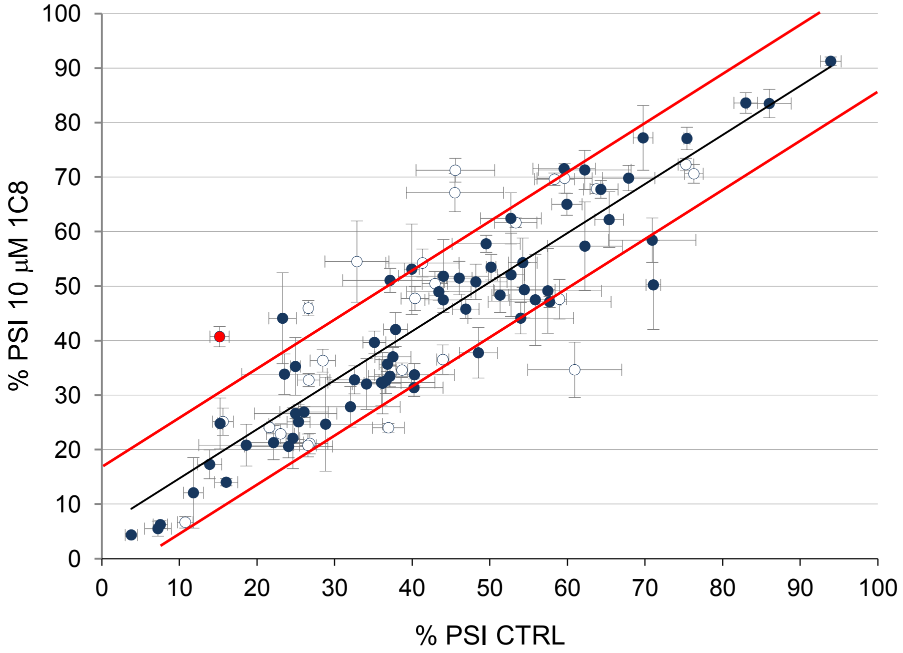

Supplementary Figure S3

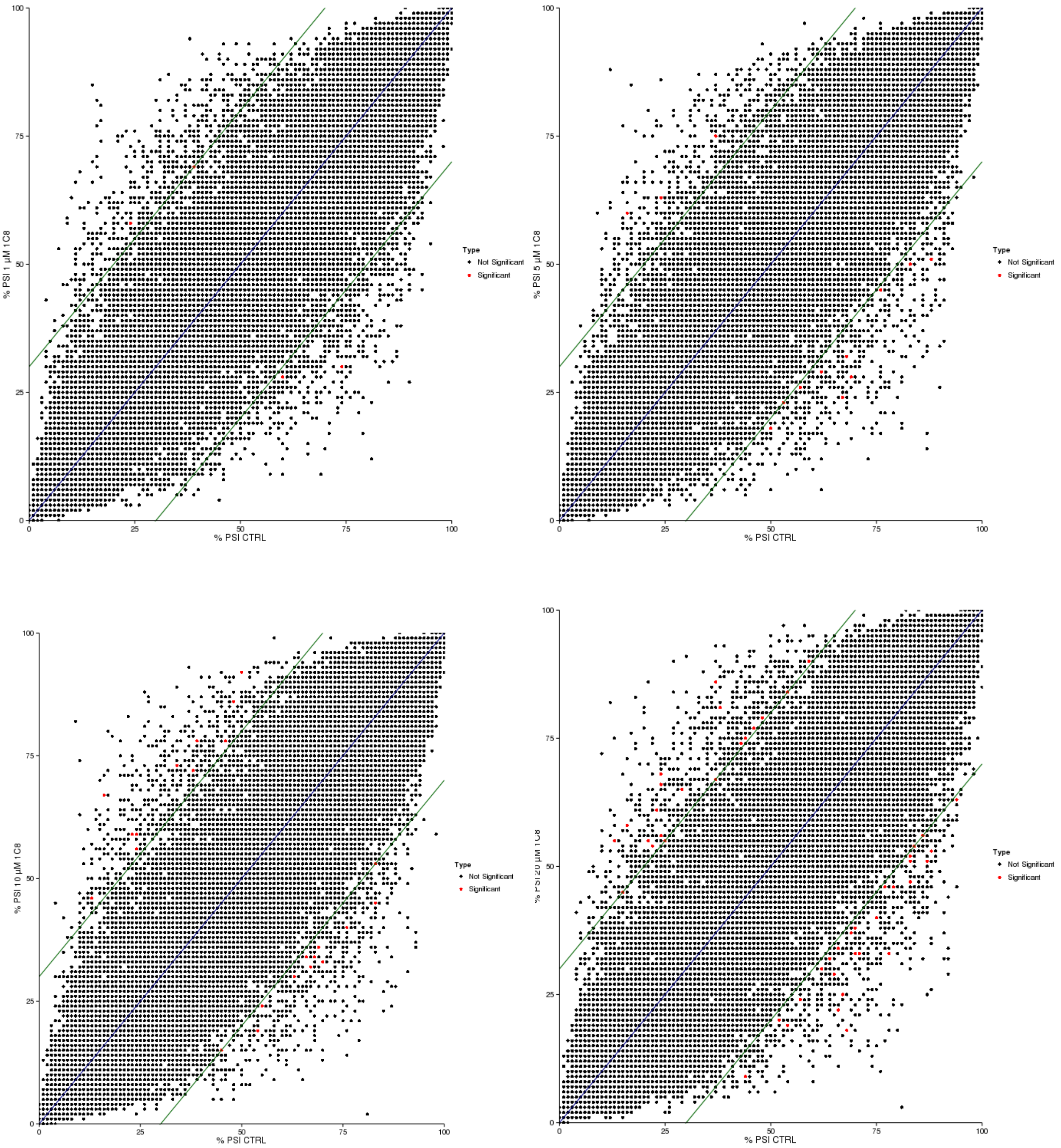

Supplementary Figure S4

A

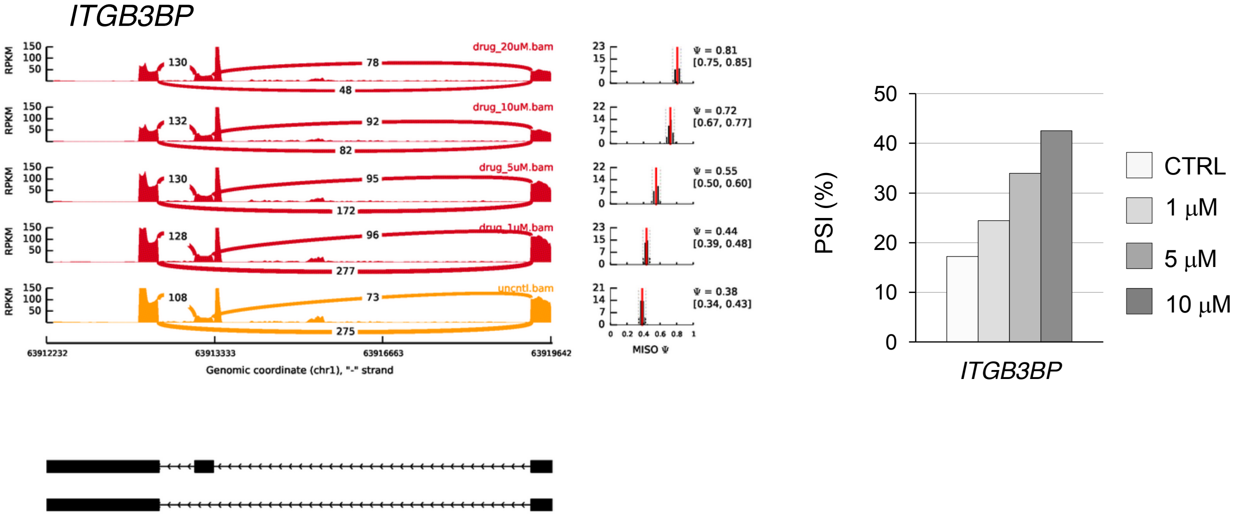

B

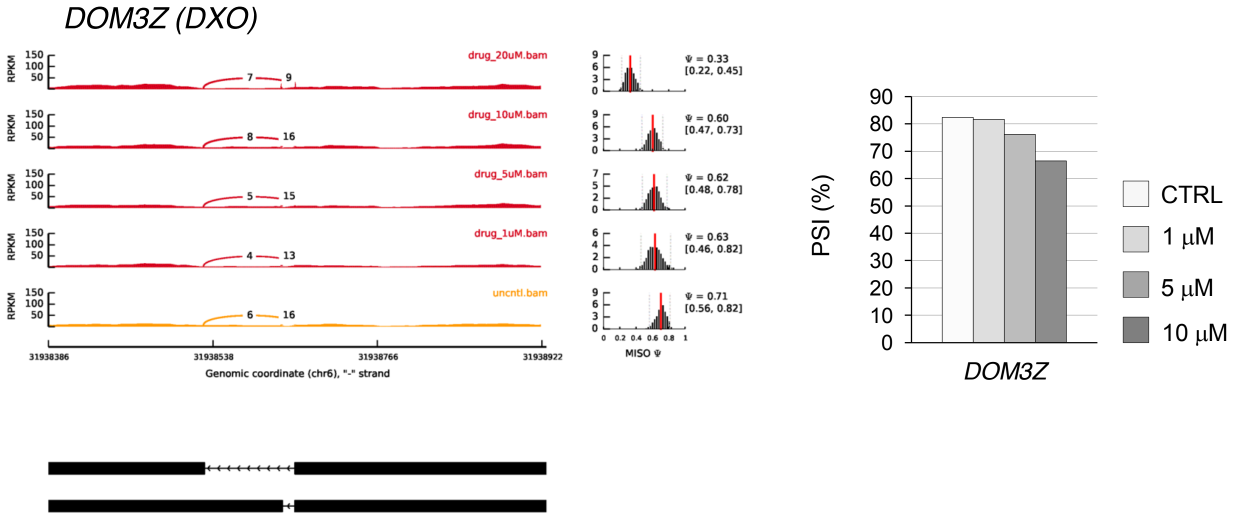

C

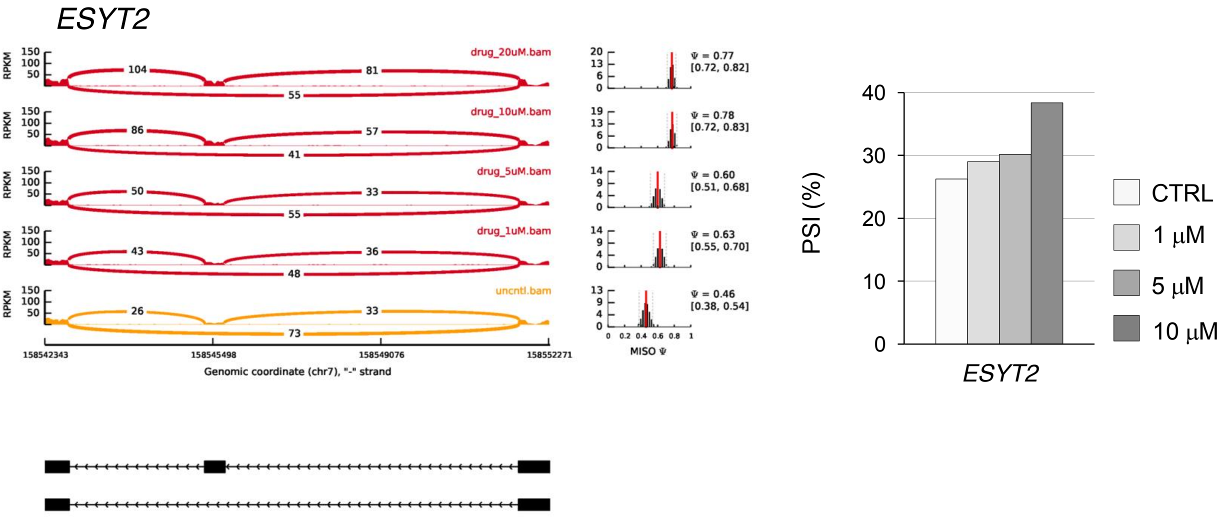

Supplementary Figure S5

D

RBM41

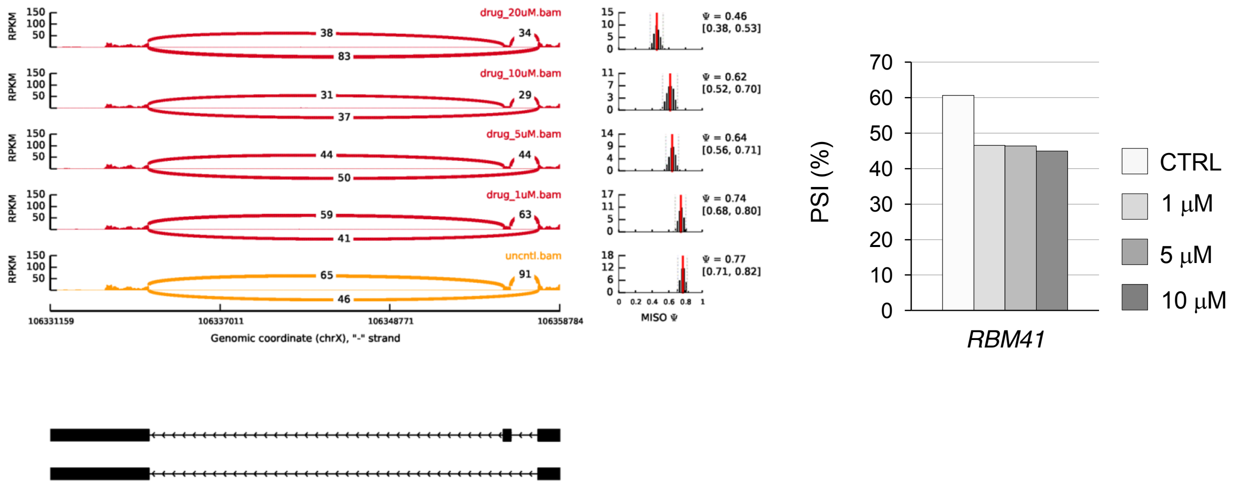

E

SPTAN1

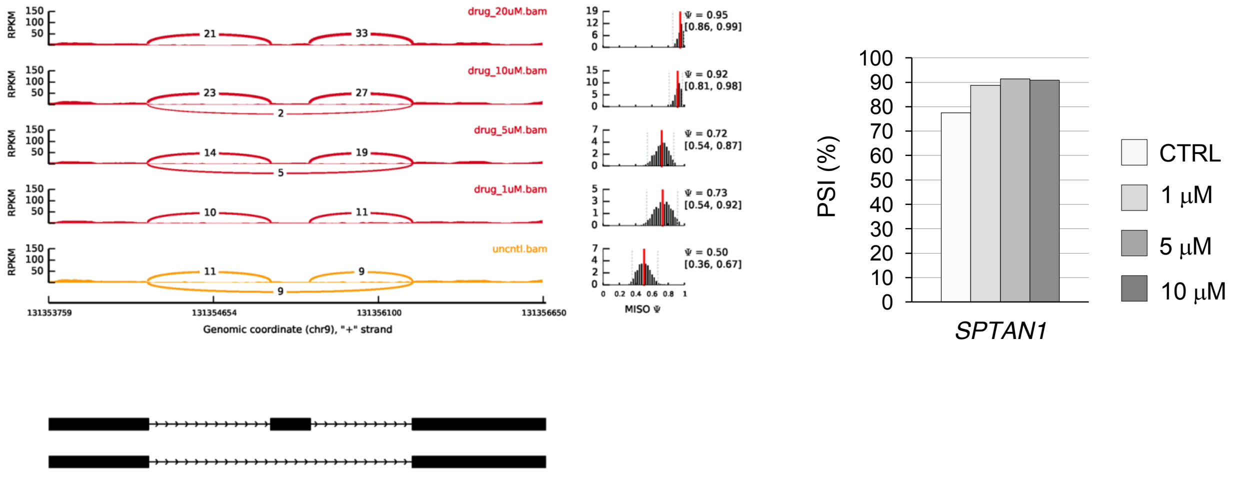

Supplementary Figure S5

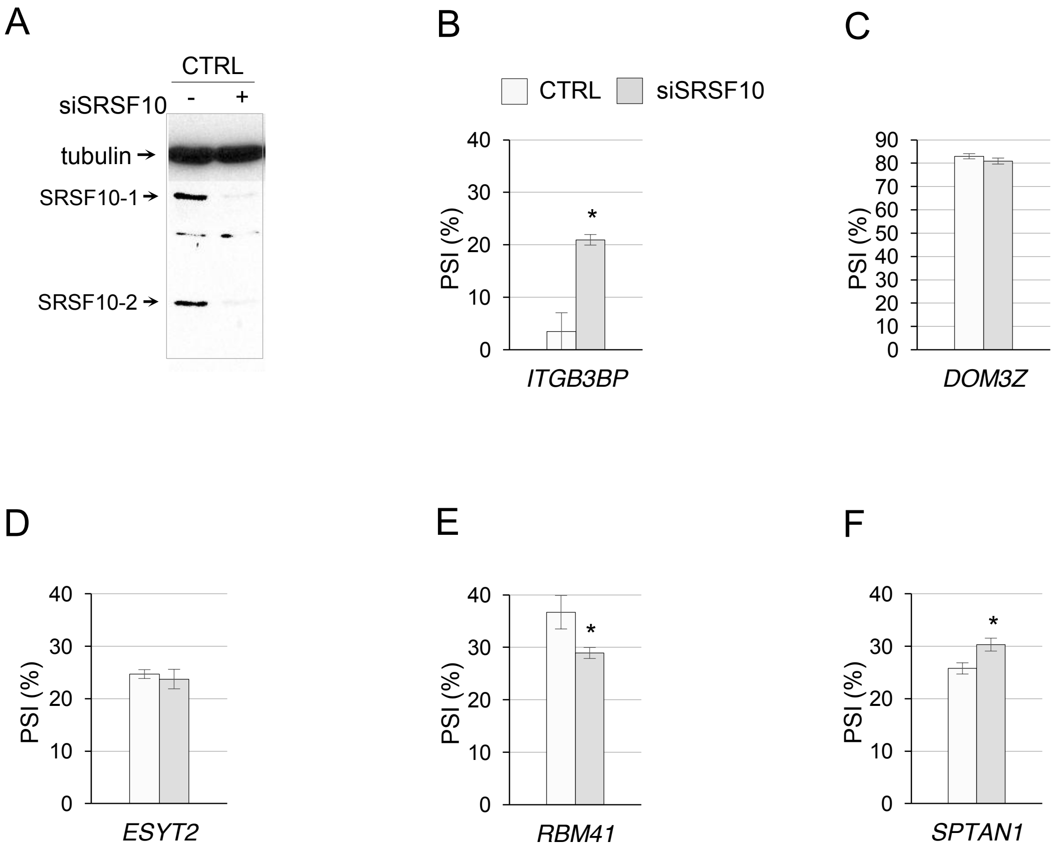

Supplementary Figure S6

A

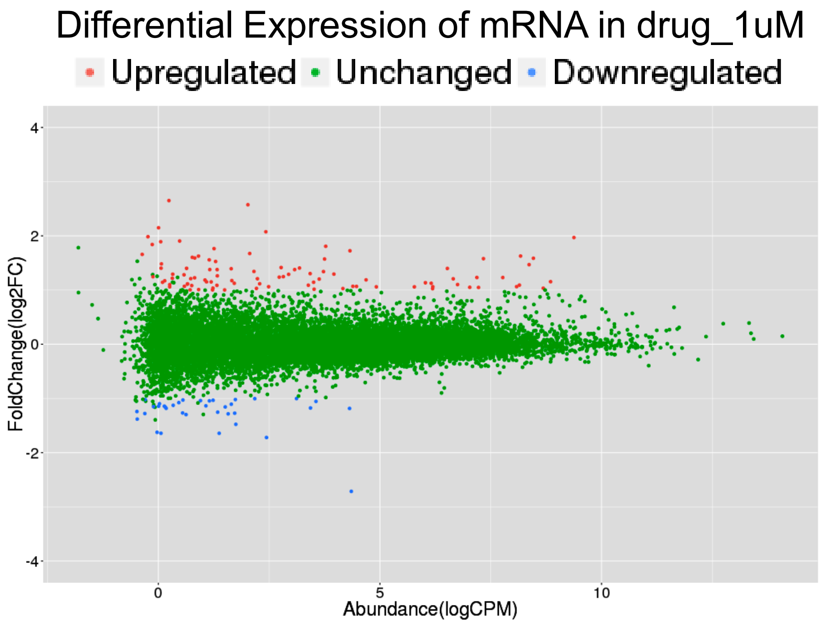

B

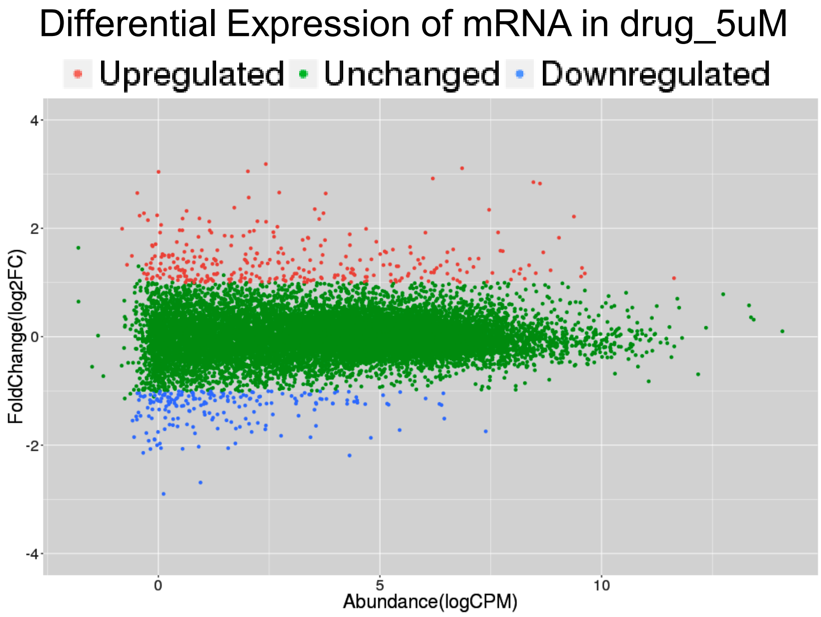

Supplementary Figure S7

C

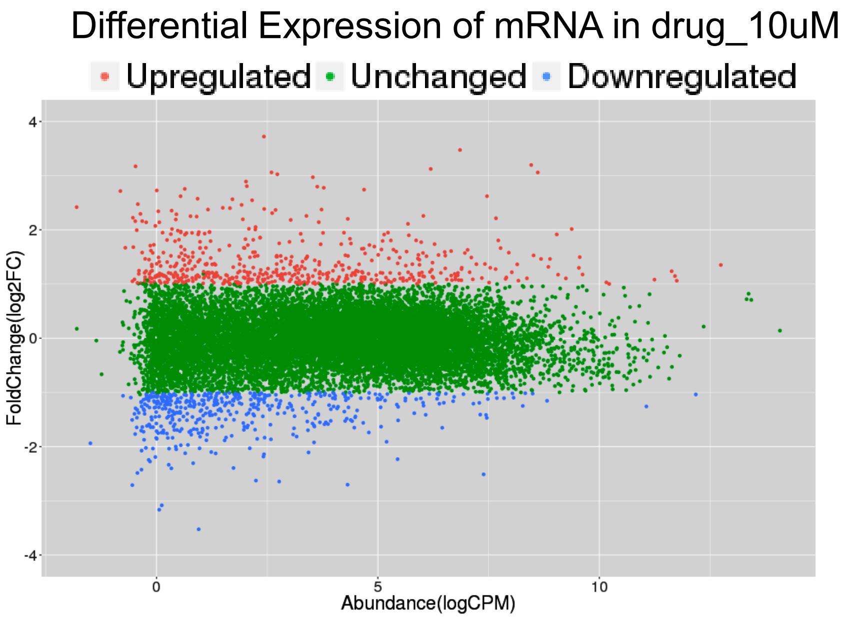

D

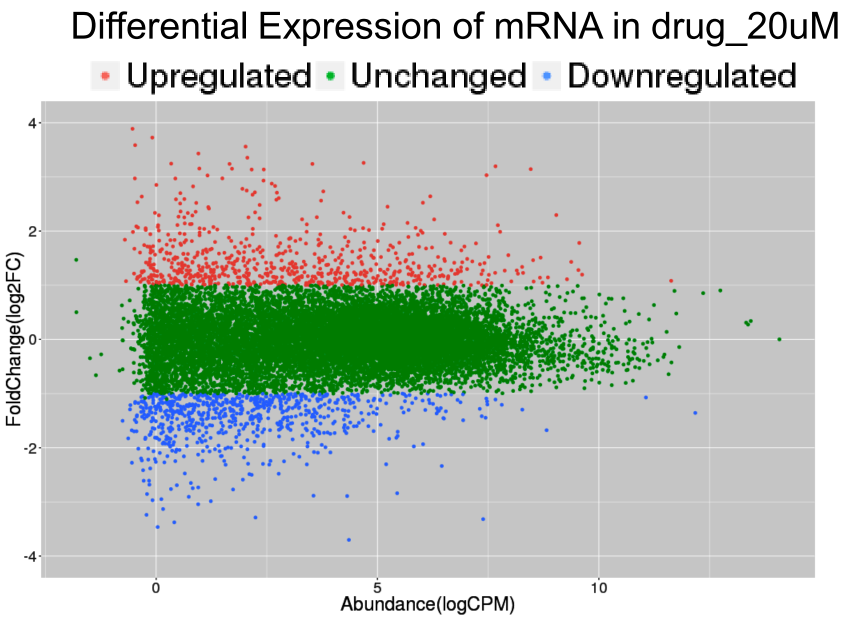

Supplementary Figure S7

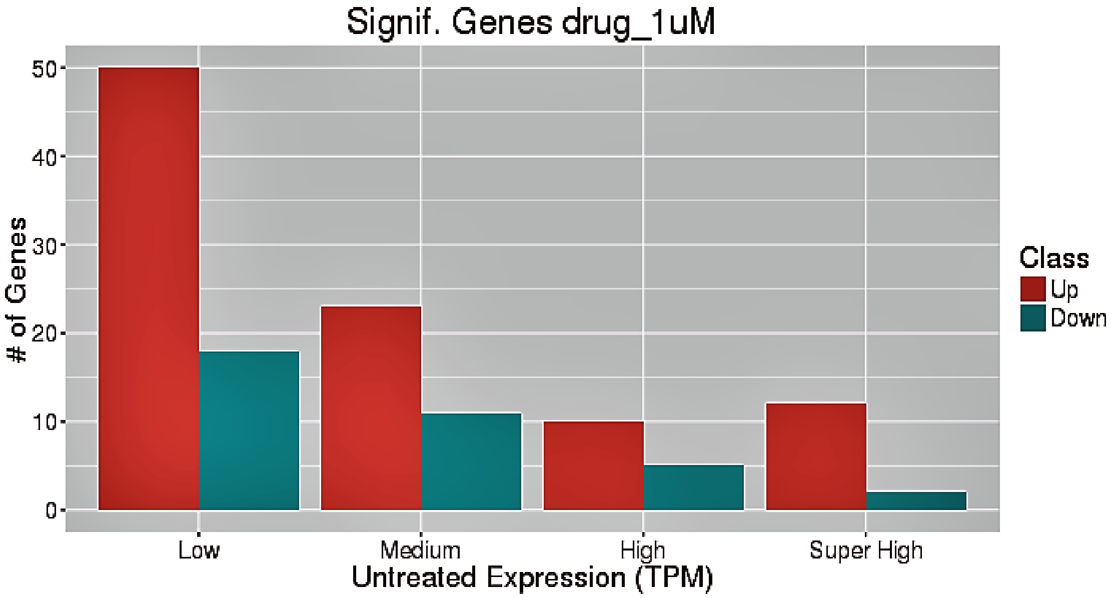

Supplementary Figure S8
